# Lumped parameter modeling of changes in liver hemodynamics due to cirrhosis

**DOI:** 10.1007/s10237-026-02055-9

**Published:** 2026-05-22

**Authors:** Edith Luveina Joseph, Himanshi Saini, Usha Kini, Timothy L. Pruett, Joseph Sushil Rao, Jeffrey Tithof

**Affiliations:** 1 Department of Mechanical Engineering, University of Minnesota, 111 Church St SE, Minneapolis, Minnesota 55455 USA; 2 Department of Pathology, St. John’s National Academy of Health Sciences, Bangalore, Karnataka 560034 India; 3 Division of Solid Organ Transplantation, Department of Surgery, University of Minnesota, Minneapolis, Minnesota 55455 USA; 4 Schulze Diabetes Institute, Department of Surgery, University of Minnesota, 420 Delaware St SE, Minneapolis, Minnesota 55455 USA

**Keywords:** Hepatic blood flow, Cirrhosis, Reduced order modeling, Computational fluid dynamics

## Abstract

This numerical study employs a computational model of human hepatic blood flow to investigate the hemodynamic consequences of cirrhosis. The liver receives blood through a dual-inlet system (portal vein and hepatic artery) that perfuses a complex network of sinusoids; however, cirrhosis-induced fibrosis distorts these channels, increasing hydraulic resistance. In turn, portal pressure is elevated, leading to the potential recanalization of portosystemic collaterals. We extended a lumped parameter hydraulic network model of the human liver, with demarcation of Couinaud segments, to capture the effects of fibrosis by incorporating a portosystemic collateral pathway and an “apparent viscosity” formulation that accounts for the non-Newtonian properties of blood. Our simulations indicate that segment-wise reductions in mean sinusoid conductance, combined with increased heterogeneity, drive the flow redistribution characteristic of the clinical “atrophy-hypertrophy complex”. This results in diminished perfusion and wall shear stress in right-lobe segments, while flow and wall shear stress is preserved or even elevated in the left lobe. Sensitivity analysis suggests that the onset of portal hypertension is driven primarily by the reduction of mean sinusoid conductance, not increased heterogeneity. However, increased heterogeneity likely exacerbates liver dysfunction, as the model predicts that a disproportionately large fraction of blood passes through the relatively few sinusoids with the lowest hydraulic resistance. Additionally, results demonstrate that while the dilatation of portosystemic collaterals effectively offloads the portal system, it leads to a substantial increase in overall blood flow, contributing to hyperdynamic circulation (a hallmark feature of cirrhosis). These predictions provide quantitative, mechanistic insights into the hemodynamic and anatomical alterations of cirrhosis, offering a computational framework that helps explain clinical observations and may assist in future patient-specific surgical planning.

**Supplementary Information:**

The online version contains supplementary material available at 10.1007/s10237-026-02055-9.

## Introduction

The liver is the largest solid organ in the body, accounting for about 2.5% of the human adult body weight, and it performs more than 500 identified vital functions (Ozougwu 2017). One of the primary functions of the liver is blood filtration, through which the liver breaks down drugs, alcohol, bacteria, and other harmful substances. To facilitate this filtration, blood from the stomach and intestines flows to the liver via the portal vein. In addition, oxygen-saturated blood is also supplied to the liver via the hepatic artery. These two parallel inlet vessels branch over several generations and eventually merge at the microvascular level, where they feed blood into a complex network of branching channels throughout the liver known as sinusoids. Blood then drains out from the sinusoids to the hepatic vein, which merges over several generations and eventually drains into the vena cava. Long-term impairment of hepatic filtration of blood may ultimately lead to chronic liver disease.

**Fig. 1:**
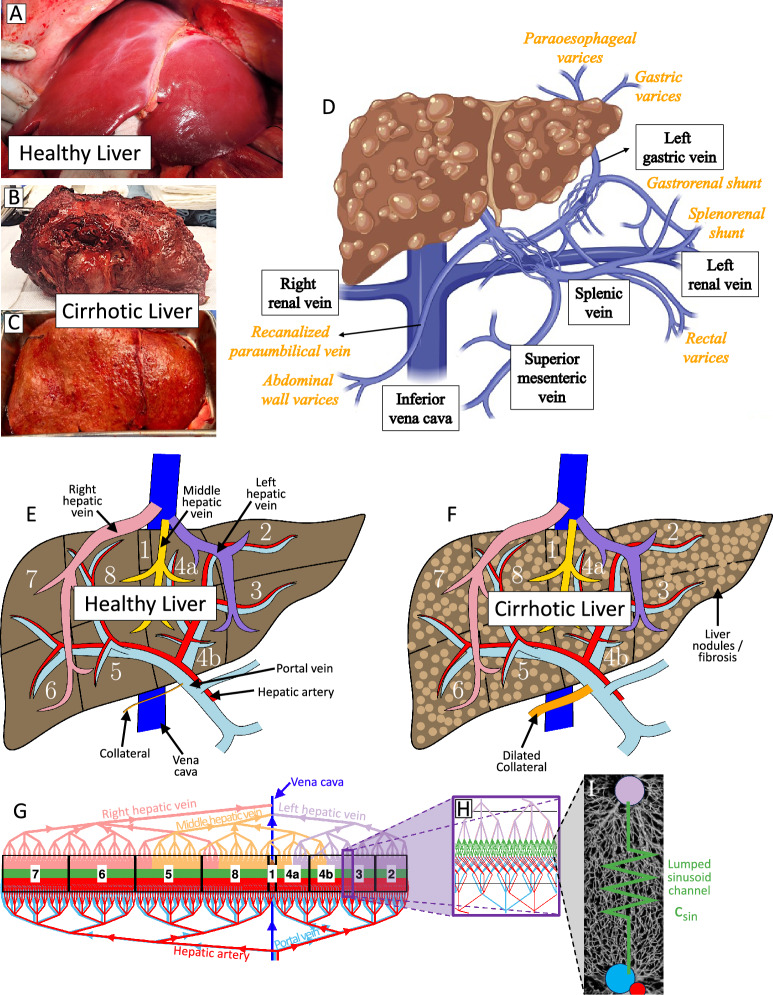
Clinical images and qualitative schematics depicting hepatic alterations due to cirrhosis. **A**–**C** Gross appearance of normal and cirrhotic livers. While **A** a normal liver is well-perfused with a smooth surface and sharp edges on the left and right lobes, the **B** under surface and **C** anterior surface of a cirrhotic liver exhibits an irregular surface with multiple fibrotic firm nodules. Fatty change of the liver, a classic feature of cirrhosis, is also apparent. **D** Schematic detailing vasculature upstream of the portal vein; varices, shunts, and collaterals are indicated in orange. **E-I** Schematic of the model of blood flow through the human liver, including **E** an illustration of healthy liver anatomy including the inflow and outflow vasculature for each liver segment and a healthy collateral, and **F** an illustration of the anatomy of the cirrhotic liver, including fibrosis and a dilated collateral. Note that the single collateral is intended to model the combined hydraulic conductance of the varices, shunts, and collaterals shown in **D**. **G** Visual representation of the numerical model with boxes corresponding to each liver segment. The size of the box is proportional to the liver segment volume fraction. Note that blood outflow from segment 1 drains directly into the vena cava, is plotted in brown, and is barely visible. Also, this is a qualitative schematic of the branching tree with a splitting number of 3 for most of the branches. The true model uses a heterogeneous splitting number taken from Debbaut et al. (2011). **H** Enlarged view of the idealized geometry showing each portal triad is connected to central (hepatic) veins via three lumped sinusoid channels (green). **I** *In vivo* image of the liver sinusoid network reported by Ishikawa et al. (2021) (gray) with an overlaid equivalent hydraulic resistor which we refer to as a “lumped sinusoid channel” (green). This channel has conductance $c_{sin}$ and is varied to model changes that occur in cirrhosis, including fibrotic matrix deposition, distortion of the microvascular architecture, hepatocyte swelling, and inflammation

Chronic liver disease (CLD) affects an estimated 1.5 billion people worldwide (Moon et al. 2020), and associated complications account for about 3.5% of all deaths globally (Ryerson et al. 2016). In particular, CLD may culminate in liver cirrhosis, which refers to an advanced stage of liver pathology characterized by inflammation, fibrosis, and cellular degeneration (Fig. 1A-C). These alterations impair liver filtration of blood, cause portal hypertension, and may lead to hepatocellular carcinoma (liver cancer), potentially culminating in liver failure and death (Schuppan and Afdhal 2008). Advances in diagnosis and treatment of cirrhosis have limited impact on long-term survival and require resource-heavy treatment such as liver transplantation (Huang et al. 2023). Additionally, the significant disproportion in supply and demand of livers (George et al. 2015) calls for early detection tools and preventative care that limits progression of liver disease to reduce the societal burden.

The progression of cirrhosis is characterized by profound changes in liver hemodynamics. As fibrosis advances, the liver sinusoids narrow and become distorted, leading to increased intrahepatic vascular resistance (Ozaki et al. 2020; Iwakiri et al. 2014). This increased resistance causes portal hypertension, which may lead to development of portosystemic collaterals through the progressive opening of preexisting vessels. Portosystemic collaterals refer to preexisting, unused vessels (e.g., the paraumbilical vein) that directly connect the hepatic portal vein to the venous systemic circulation, through which portal blood flow may be redirected to bypass the liver (Philips et al. 2016; Kang et al. 2002); see orange labels in Fig. 1D. Collateral formation also leads to additional complications such as variceal bleeding (Maruyama and Shiina 2021) and hepatic encephalopathy (Khungar and Poordad 2012). Collateral development, through the opening of preexisting vessels or via sprouting and intussusceptive angiogenesis, is an ongoing area of study (Iwakiri et al. 2014). Additionally, cross-sectional imaging utilizing CT and 4D flow MRI has linked these hemodynamic shifts to distinct morphological patterns. Studies demonstrate that reduced right portal vein flow is associated with right-lobe atrophy, while compensatory increases in flow drive the hypertrophy of the left and caudate lobes, where sinusoidal and portal venous pathways remain relatively preserved (Harbin et al. 1980; Higaki et al. 2023).

The numerical modeling described in this work builds on an extensive literature in which a variety of computational models and medical imaging techniques have been leveraged to model hepatic flow in a variety of hepatic diseases. Wang et al. (2017) used a coupled 0D model of hepatic circulation, with a 1D model of the cardiovascular system to study the sensitivity of the hepatic venous pressure gradient to vascular changes due to cirrhosis. Peeters et al. (2015) generated surface meshes from radiological imaging of a corrosion cast of a cirrhotic human liver; they then performed three-dimensional (3D) simulations to investigate microcirculatory flow, which they found to be nonhomogeneous and anisotropic. Yoshizawa et al. (2022) and Dutta-Moscato et al. (2014) each used agent-based models of liver tissue to study liver fibrosis dynamics and fibrosis progression, respectively. George et al. (2015) used magnetic resonance imaging and 3D simulations to study hemodynamics of cirrhotic human livers; they quantified the fraction of blood that flows from the superior mesenteric vein to each of the right and left liver lobes.

Here, we have further developed an existing computational model (Tithof et al. 2023) with the goal of predicting fluid mechanical aspects of the progression of cirrhosis. Since the human hepatic vasculature branches over several generations, the total number of vascular segments exceeds tens of millions (Debbaut et al. 2011). It is therefore computationally intractable to simulate the entire liver vasculature with full 3D resolution. However, lumped parameter modeling—which forms the basis of our approach—is much more computationally tractable, enabling simulation of massive vasculature networks on a standard laptop in minutes (Tithof et al. 2023). Lumped parameter modeling involves solving the hydraulic analog of Ohm’s law, $\Delta p = Q R$, where $\Delta p$ is the pressure drop over a vessel segment, *Q* is the volume flow rate, and *R* is the hydraulic resistance. This approach has been used extensively in the past for modeling hepatic blood flow. Van Der Plaats et al. (2004) modeled the entire hepatic vascular tree of the dog liver and reported good agreement in the computed and experimental pressure and velocity. Debbaut et al. (2011, 2014) characterized statistics of the branching human liver vasculature via corrosion casting; their measurements formed the basis for simulations which led to novel insights into internal pressure and flow distributions during hypothermic machine perfusion, as well as the functional organization of the liver vasculature. Audebert et al. (2017) developed a closed-loop lumped model to study hemodynamic changes during liver ablation surgery in pigs and found that model predictions agreed well with experimental values. More recently, Torres Rojas et al. (2021) combined dendritic vascular trees with a porous medium model of microvasculature to study the effects of thrombosis.

In this article, we present simulations in which we have extended an existing lumped parameter hydraulic network model of blood flow through the entire human liver (Tithof et al. 2023) to study the effects of cirrhosis on liver hemodynamics (Fig. 1E–F). The original model includes realistic heterogeneities in vascular branching and incorporation of model parameters obtained from clinical measurements of decreased donor livers. Novel features of the extended model presented here include the addition of a collateral pathway with variable conductance, viscosity correction to account for non-Newtonian behavior of blood and a revised implementation of the boundary conditions to more accurately model the progression of cirrhosis. We also propose a model for early and advanced cirrhosis by varying sinusoid conductance and heterogeneity segment-wise to reflect atrophy/hypertrophy complex.

Our model provides significant insights with potential for future applications in personalized medicine, which also improves our fundamental understanding of cirrhosis by characterizing the complex interplay of alterations in sinusoid anatomy (due to fibrotic matrix deposition, distortion of the microvascular architecture, hepatocyte swelling, and inflammation) leading to greater sinusoid heterogeneity and increased hydraulic resistance, as well as portosystemic collateral dilation and portal hypertension. We characterize two important hemodynamic-related features of cirrhosis which very likely contribute to impairment of critical liver function: (i) heterogeneous liver perfusion which directs a large proportion of hepatic blood flow through a small fraction of the liver sinusoids and (ii) shunting of blood away from the liver to collateral pathways that bypass the liver. In the future, these aspects of the model can be adapted to capture anatomy of individual patients to both guide and predict the success of various clinical interventions. In addition, our model helps explain clinical observations during cirrhosis.

This article is organized as follows: Section 2 details the hydraulic network model methodology, including recent improvements and extensions. Results are presented and discussed in Sect. 3. Finally, conclusions and possibilities for future work are presented in Sect. 4.

## Methods

Simulations presented here build on an existing lumped parameter hydraulic network model of hepatic blood flow (Tithof et al. 2023). The model is organized into “edges” and “nodes,” where an “edge” refers to a blood-carrying vessel with prescribed length and diameter, and a “node” refers to each point at which a vessel branches or merges with another vessel. A visual representation of the model (with very few generations, for the sake of clarity) is plotted in Fig. 1G. The model consists of inflow from the hepatic artery (red) and the portal vein (light blue), which branches across several generations, eventually reaching lumped sinusoid channels, as illustrated in Fig. 1H–I. The physical interpretation of the lumped sinusoid channels, and how they are altered as cirrhosis progresses, is discussed below. Blood drains from the lumped sinusoid channels into the centrilobular veins, which merge over several generations to form the right hepatic vein (RHV), middle hepatic vein (MHV), and the left hepatic vein (LHV), eventually draining into the vena cava (dark blue). Our model also includes a collateral pathway (not plotted in Fig. 1G, but illustrated in Fig. 1D–F) which shunts blood away from the liver. Observation of a “short-circuit” pathway is common in cirrhosis (Kurosaki et al. 1994), and progressive opening of such vessels in response to increases in portal pressure is well established (Nardelli et al. 2020).

### Model parameters

The parameters used in our hydraulic network model are given in Table 1, 9 of which were obtained from literature and 5 of which were clinically measured from deceased donors (Tithof et al. 2023). An important feature of this model is that it incorporates the Couinaud segmentation, which is a common clinical scheme which labels nine different regions of the liver. Here, we use volume fractions and proportions of flow to each segment that are identical to those used in Tithof et al. (2023) for the healthy liver. All simulations assume a mean pressure difference of 77 mmHg for the hepatic artery (relative to the vena cava). Simulations of a healthy liver assume that the pressure difference at the inlet of the portal vein is 5 mmHg (relative to the vena cava) (Ricken et al. 2010). The splitting numbers define the mean number of daughter vessels at each branching point as the hepatic artery, portal vein, and hepatic vein approach their terminal vessels. The liver vasculature networks we develop are randomly generated, with the number of daughter vessels at a given node determined by randomly sampling from a normal distribution with a user-specified mean and standard deviation, given by the splitting numbers in Table 1 which come from Debbaut et al. (2011) and are based on human liver corrosion casting. (The splitting number at a given node is an integer determined by rounding each floating point randomly generated number.) The number of branching generations for each vascular tree is computed as $g = [\log N/\log s]$, where *N* and *s* are the number of terminal vessels and splitting number for a given vascular tree, respectively, and $[\cdot ]$ denotes rounding to the nearest integer. The multiplicative change in vessel radius $\gamma$ across generations is then computed from $r_f=\gamma ^g r_0$, where $r_0$ is the base radius and $r_f$ is the terminal radius, listed in Table 1 for all three vascular trees. Finally, the length ratio specifies the length of each segment between sequential branching points as a constant multiple of the vessel diameter. More details are provided in Tithof et al. (2023).

**Table 1:** Parameters used in the numerical model.

Parameter	Value
Hepatic Artery Inlet Pressure Difference	77 mmHg
Portal Vein Inlet Pressure Difference (Initial Healthy Simulation)	5 mmHg
Liver Volume	1518 cm$^3$ (Abdalla et al. 2004)
Splitting Number of Hepatic Artery	2.76 ± 1.01 (Debbaut et al. 2011, 2014; Lorente et al. 2020)
Splitting Number of Portal Vein	2.80 ± 0.61 (Debbaut et al. 2011, 2014; Lorente et al. 2020)
Splitting Number of Hepatic Vein	3.11 ± 1.46 (Debbaut et al. 2011, 2014; Lorente et al. 2020)
Length Ratio of Hepatic Artery	4.85 (Kline et al. 2011)
Length Ratio of Portal Vein	5.11 (Kline et al. 2011)
Length Ratio of Hepatic Vein	5.00
Hepatic Artery Base Lumen Radius	2.2 mm (Tithof et al. 2023)
Portal Vein Base Lumen Radius (Healthy)	4.9 mm (Tithof et al. 2023)
Portal Vein Base Lumen Radius (Early Cirrhosis)	6.4 mm
Portal Vein Base Lumen Radius (Advanced Cirrhosis)	7.2 mm
RHV Base Lumen Radius	11.7 mm (Tithof et al. 2023)
MHV Base Lumen Radius	4.0 mm (Tithof et al. 2023)
LHV Base Lumen Radius	6.5 mm (Tithof et al. 2023)
Hepatic Artery Terminal Lumen Radius (Bayesian optimization)	2.61 $\mu$m
Portal Vein Terminal Lumen Radius (Bayesian optimization)	67.5 $\mu$m
Hepatic Vein Terminal Lumen Radius (Bayesian optimization)	75.3 $\mu$m

Pressure differences are relative to the vena cava pressure

**Table 2:** Liver segment volumes (cm$^3$) used for simulations.

Segment	Healthy and early cirrhosis simulations (Abdalla et al. 2004)	Advanced cirrhosis simulations
Segment I	28	77
Segments II–III	242	341
Segments IV–VIII	1248	1035

The healthy and early cirrhosis values are from Abdalla et al. (2004). The advanced cirrhosis values were calculated by applying the fold change between healthy and cirrhotic mean volumes reported by Hunt et al. (2016) to the Abdalla et al. baseline data.

In the progression of cirrhosis, the portal vein (PV) diameter is altered. A study by Yazdi and Khalilian (2005) compared the mean PV diameter in healthy controls and cirrhotic patients. They reported a mean PV diameter of 8.9 mm in the healthy group and 11.6 mm in the patient group, representing an approximately 30% increase for those with cirrhosis. The study also evaluated a PV diameter threshold of $>13$ mm, which corresponds to an approximate $50\%$ increase from the reported healthy mean, though this threshold was found to have high specificity but low sensitivity for diagnosing cirrhosis. While this study did not distinguish disease severity of patients (e.g., “early” vs. “advanced”), it did confirm a significant trend of portal venous dilatation. Guided by this reported trend, the PV diameters used in this study to model anatomy of healthy patients and those with cirrhosis is given in Table 1. Moderate cirrhosis is modeled using a 30% larger diameter and the advanced case is modeled with a 50% larger diameter than that of the healthy baseline.

The progression of cirrhosis induces significant morphological changes in the liver, characterized by a redistribution of segmental volumes. The volumetric parameters for the liver segments in the healthy and early cirrhosis simulations were adopted from the study by Abdalla et al. (2004), as detailed in Table 2. To establish the parameters for the advanced cirrhosis model, these baseline volumes were adjusted using the fold change between healthy and cirrhotic mean volumes reported by Hunt et al. (2016). This calculation method simulates the characteristic morphological changes of advanced cirrhosis, namely the hypertrophy of Segment I and Segments II–III, and the atrophy of Segments IV–VIII.

### Bayesian parameter estimation

In the liver vasculature, the terminal vessels of the portal vein and hepatic artery merge to feed blood into a network of sinusoids, which then drain into a centrilobular vein (Fig. 1I). For computational efficiency, we simplify this complex anatomical structure into a single “lumped sinusoid channel” with a conductance $c_{sin}$ that is varied as cirrhosis progresses.

To calibrate the model for a healthy individual, we used Bayesian optimization to determine the optimal combination of $c_{sin}$ and the terminal HA, PV, and HV lumen diameters. The optimization was implemented using MATLAB’s “bayesopt” function, run for 50 iterations, and constrained by physiologically plausible diameter ranges for the terminal vessels as reported in the literature (Crawford et al. 1998). The objective function was formulated to minimize the total error between the model’s predicted flow rates and the clinically measured target: a total liver volume flow of 1300 ml/min, with the portal vein supplying about 75% of this flow (970 ml/min) (Brunsing et al. 2021).

This optimization yielded the set of terminal vessel lumen diameters listed in Table 1 and an optimal sinusoid conductance $c_{sin} = 1.2\times 10^{-5}$ mmHg$\cdot$min/ml. With these parameters, our model successfully reproduces the target clinical flow rates, confirming that the hydraulic conductance of our lumped channel is a valid representation of a healthy local sinusoid network. To model the progression of cirrhosis, we then vary the lumped sinusoid channel conductance $c_{sin}$, a control parameter calculated from the sinusoid diameter *d* as detailed in the next section.

### Governing equations and boundary conditions

All simulations presented here contain a total of 5 million lobules (each containing six lumped sinusoid channels), and the total number of edges in a given simulation is about 68 million; further details on the model geometry are available in Tithof et al. (2023). Each edge (i.e., vascular segment) connecting nodes *i* and *j* has a hydraulic conductance (inverse of hydraulic resistance) given by:

1 $$\begin{aligned} \begin{gathered} c_{ij}=\frac{\pi d^4}{128 \mu L}, \end{gathered} \end{aligned}$$

where *d* is the vessel diameter, $\mu$ is the dynamic viscosity, and *L* is the vessel segment length. Laminar Poiseuille flow was used since all flows are at small Reynolds numbers (Tithof et al. 2023). The associated linear algebra describing this massive network of interconnected hydraulic resistors is of the form $Cp = z$ given by:

2 $$\begin{aligned}&\begin{bmatrix} c_{1,2} & -c_{1,2} & 0 & \dots & 0 & -1 & 0 \\ -c_{1,2} & c_{1,2}+c_{2,3} & -c_{2,3} & \dots & 0 & 0 & 0 \\ 0 & -c_{2,3} & \ddots & \dots & 0 & 0 & 0 \\ \vdots & \vdots & \vdots & \vdots & \vdots & \vdots \\ 0 & 0 & 0 & \dots & c_{n-1,n} & 0 & 0 \\ 1 & 0 & 0 & \dots & -1 & 0 & 0 \\ 0 & 0 & 0 & \dots & -1 & 0 & 0 \\ \end{bmatrix} \begin{bmatrix} p_1 \\ p_2 \\ p_3 \\ \vdots \\ p_n \\ Q_{HA} \\ Q_{PV} \\ \end{bmatrix} \nonumber \\ &\quad = \begin{bmatrix} 0 \\ 0 \\ 0 \\ \vdots \\ 0 \\ \Delta p_{HA} \\ \Delta p_{PV} \\ \end{bmatrix} \end{aligned}$$

where *C* is a sparse matrix containing conductance values $c_{i,j}$, $p_j$ are the unknown values of pressure at each node in the network, *m* is the total number of nodes forming the hepatic artery, *n* is the total number of nodes in the network, *z* is entirely zeros except for the last two entries $\Delta p_{HA}$ and $\Delta p_{PV}$, which specify the hepatic artery and portal vein inlet pressure gradients, respectively, and $Q_{HA}$ and $Q_{PV}$ are the unknown total hepatic artery and portal vein volume flow rates, respectively. The volume flow rate through every vessel segment is computed as $Q_{i,j}= c_{i,j} ( p_i - p_j)$. Each row in linear algebra system (2) corresponds to enforcing the continuity equation at a particular node in the hydraulic network. For example, the first row of Eq. (2) is written as:

3 $$\begin{aligned} c_{1,2}(p_1-p_2)-Q_{HA}=0. \end{aligned}$$

This linear algebra system was solved for a healthy liver (i.e., $c_{sin} = 1.2\times 10^{-5}$ mmHg$\cdot$min/ml and parameters from Table 1), and all standard volume flow rates were stored. Wall shear stress in each vessel was calculated as:

4 $$\begin{aligned} \begin{gathered} WSS_{ij}=U_{i,j}\, \left( \frac{32\pi \mu ^3}{ L c_{i,j}} \right) ^{1/4}, \end{gathered} \end{aligned}$$

where the average velocity $U_{i,j}$ was computed as $Q_{i,j}$ divided by the cross-sectional area of each given vessel. An example linear algebra system for a small, idealized network is provided in Section 1 of the Supplementary Information.

Physiologically, inflow through the portal vein is not driven by a fixed pressure boundary condition, but rather it is more accurately modeled as a fixed volume flow rate boundary condition. This is because portal blood flow comes from the superior mesenteric vein which drains from the gut (including intestines, pancreas, and stomach) and the splenic vein. The gut does not possess any mechanism for regulating blood flow in response to increased hepatic resistance, and all blood from these two veins must drain into the portal vein. Hence, we prescribe a fixed volume flow rate for the portal vein for all cirrhosis cases, equal to 820 ml/min reported in Brunsing et al. (2021) for 19 patients with cirrhosis, which is about 15% lower than that of the mean of healthy individuals, and we compute the associated pressure. We then characterize the extent of portal hypertension (by calculating $\Delta p_{pv}$) that arises as a consequence of alterations in sinusoidal anatomy, as well as the effects of collateral dilatation. The volume flow rate through the portal vein (820 ml/min) was then implemented as a constant volume flow rate boundary condition into the portal vein for all cirrhotic simulations (described in more detail below). Implementation of a fixed volume flow rate for the portal vein, modeling the cirrhotic liver, is achieved by replacing the pressure boundary condition given in the last row of Eq. (2) by $c_{m+1,m+2}(p_{m+1}-p_{m+2})=Q_{PV}$ where $m+1$ and $m+2$ are the start and end nodes of the inlet portal vein branch, which leads to a modified linear algebra system given by:

5 $$\begin{aligned}&\begin{bmatrix} c_{1,2} & -c_{1,2} & 0 & \dots & \dots & \dots & \dots & 0 & -1 & 0 \\ -c_{1,2} & c_{1,2}+c_{2,3} & -c_{2,3} & \dots & \dots & \dots & \dots & 0 & 0 & 0 \\ 0 & -c_{2,3} & \ddots & \dots & \dots & \dots & \dots & 0 & 0 & 0 \\ \vdots & \vdots & \vdots & \vdots & \vdots & \vdots & \vdots & \vdots & \vdots & \vdots \\ 0 & 0 & 0 & \dots & \dots & \dots & \dots & c_{n-1,n} & 0 & 0 \\ 1 & 0 & 0 & \dots & \dots & \dots & \dots & -1 & 0 & 0 \\ 0 & 0 & 0 & \dots & c_{m+1,m+2} & -c_{m+1,m+2} & \dots & 0 & 0 & 0 \\ \end{bmatrix} \nonumber \\ &\quad \times \begin{bmatrix} p_1 \\ p_2 \\ p_3 \\ \vdots \\ p_n \\ Q_{HA} \\ Q_{PV+col} \\ \end{bmatrix} = \begin{bmatrix} 0 \\ 0 \\ 0 \\ \vdots \\ 0 \\ \Delta p_{HA} \\ Q_{PV} \\ \end{bmatrix} \end{aligned}$$

where $Q_{PV}$ is the known volume flow rate through the portal vein only for cirrhotic liver, $Q_{PV+col}$ is flow rate through portal vein and collateral, and the hepatic artery pressure $\Delta p_{HA}$ is enforced through the boundary conditions.

The linear algebra system given by Eq. (5) is constructed using MATLAB (The Mathworks, Inc., Version R2025b). For a typical network, the total number of nodes is more than $2 \times 10^7$, and *C* has over $(2 \times 10^7)^2 = 4 \times 10^{14}$ entries. Thus, sparse storage of *C* is critical for computational tractability. The vector *p* is solved using the backslash operator (i.e., $p = C \backslash z$). The largest reported networks model blood flow through approximately $2.8 \times 10^7$ nodes and $6.6 \times 10^7$ individual vessel segments. Associated files are of the order 3.3 GB, and associated calculations (network construction, solving for pressure, and computation of volume flow rate, speed, and wall shear stress), require 7.5 min on a Velocity Micro ProMagix HD80A Workstation with 24 dual-threaded cores and 256 GB of RAM.

### Collateral modeling

In patients with cirrhosis, multiple collateral pathways with variable degrees of dilatation often develop. We model the combined effect of the varices, shunts, and collateral pathways shown in Fig. 1D as a single lumped collateral vessel with equivalent conductance $c_{col}$. Our extended model includes a collateral channel (orange in Fig. 1F) which was implemented as an edge connecting the inlet of the portal vein to the vena cava. The collateral has inlet and outlet pressure equal to that of the base of the portal vein and vena cava, respectively, and we vary $c_{col}$, capturing the effect of collateral dilatation as cirrhosis progresses. The healthy liver simulation assumes that the collateral conductance is equal to 1% of the total conductance of the healthy liver, leading to a negligibly small fraction of blood flow through the collateral (expected for a healthy individual). The conductance of the healthy liver was calculated as $c_{liver} = Q/\Delta p$, where $c_{liver}=176$ ml/(min$\cdot$mmHg) is the conductance of the liver, $Q= 1300$ ml/min is the total volume flow rate through the liver, and $\Delta p=5$ mmHg is the difference in pressure at the inlet (base of portal vein) and outlet (vena cava). Our simulations tested five values of collateral conductance, $c_{col}=\{1.76,5.57,17.6,55.7,176\}$ ml/(min$\cdot$mmHg), which are equally spaced on a logarithmic scale.

### Proposed model for cirrhosis

**Fig. 2:**
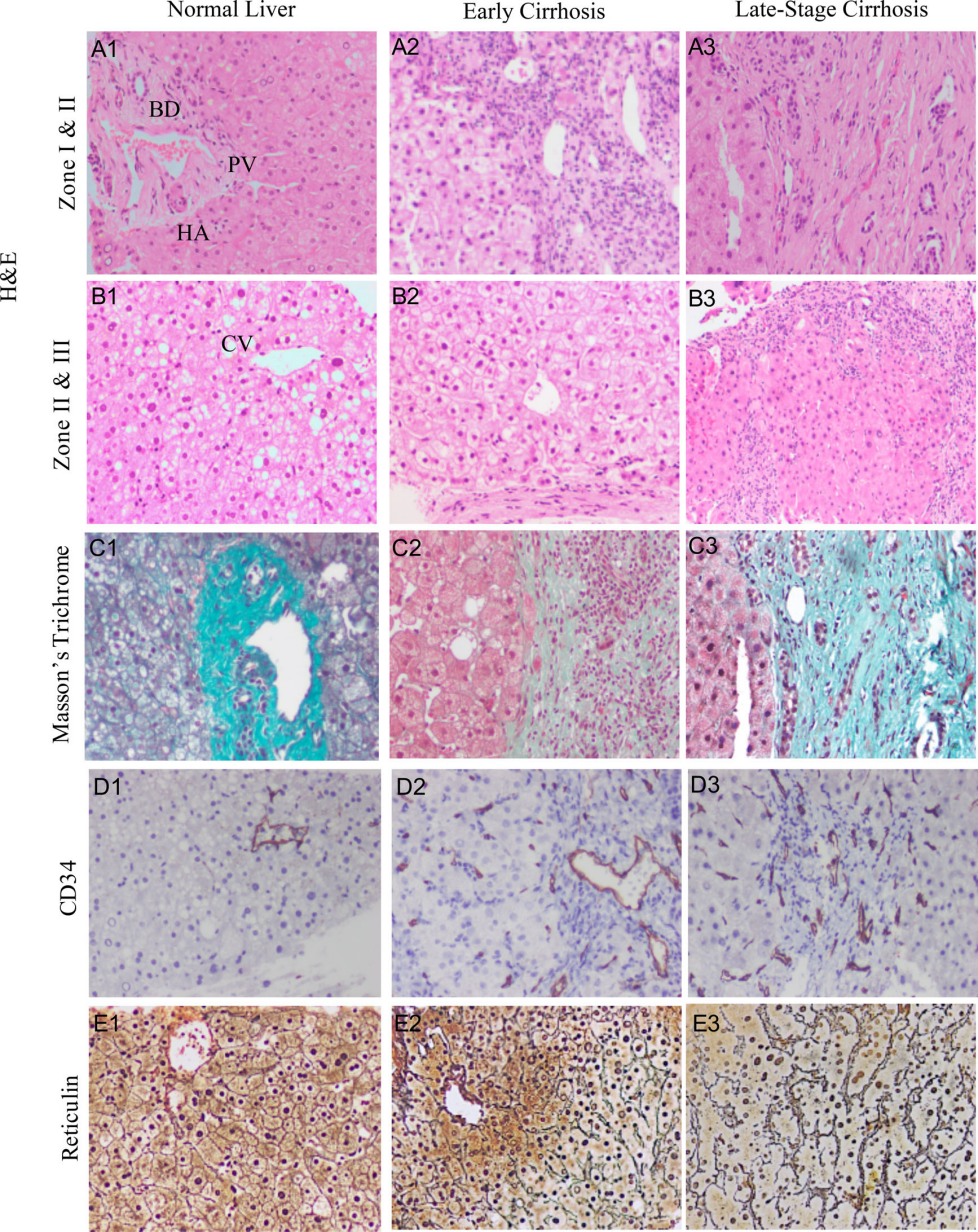
Histology of normal, early, and late-stage cirrhosis. **A**–**B** Hematoxylin and eosin (H&E) staining of Zones 1–3 demonstrate changes in cellular architecture. The normal liver (**A1**–**B1**) has nearly homogeneous hepatocyte size and shape with near normal nuclei; architecture of terminal branches of portal triad (portal vein [PV], hepatic artery [HA], and bile duct [BD]) and central vein [CV] are also normal. However, progression to early (**A2**–**B2**) or late-stage (**A3**–**B3**) cirrhosis leads to changes in liver histological architecture; the black dotted line highlights a fibrotic nodule within the parenchyma of a liver lobule with hepatocytes of irregular shape and size. **C** Masson’s Trichrome stains collagen green, non-collagenous tissue red/pink, and nuclei purple. Progressive deposition of type I collagen (mature) across early to late-stage cirrhosis leads to increasingly dense and irregular deposition of collagen. In addition, circular white spaces (**C2**) correspond to fatty change in liver parenchyma related to early-stage cirrhosis. **D** CD34 staining (brown) labeling vascular endothelial cells. For the healthy liver (**D1**), CD34 labels only the portal vein and not cells lining sinusoids. As cirrhosis progresses (**D2**–**D3**), neovascularization/capillarization/arterialization demonstrates CD34 staining of sinusoids. **E** Staining for reticulin (type III collagen) which provides a structural support for hepatocytes and sinusoids in a normal liver, but is progressively disrupted in early to advanced stage cirrhosis and replaced by mature collagen

A key component of this study is that we systematically vary the conductance of the lumped sinusoid channels $c_{sin}$ to model the microvascular changes that occur due to cirrhosis progression. To visualize these alterations, we performed histological analysis of a normal liver, an early cirrhotic liver, and an advanced cirrhotic liver (Fig. 2). These images suggest cirrhosis progression is associated with a complex interplay of sinusoidal narrowing (increasing intrahepatic hydraulic resistance) and increased heterogeneity (which may locally increase or decrease hydraulic resistance). Sinusoid narrowing may be inferred from formation of fibrotic nodules which compress adjacent tissue (Fig. 2B), as well as the progressive deposition of type I collagen (Fig. 2C). Notably, sinusoid narrowing has been linked to impaired vasodilatation (Rockey and Chung 1998; Greuter and Shah 2016) and has been directly observed in a murine model of acute liver injury (Yoon et al. 2013). Increased heterogeneity likely also arises due to progressive variation in hepatocyte size, capillarization/arterialization, and deposition of type III collagen (Fig. 2A–B, D, and E, respectively).

**Fig. 3:**
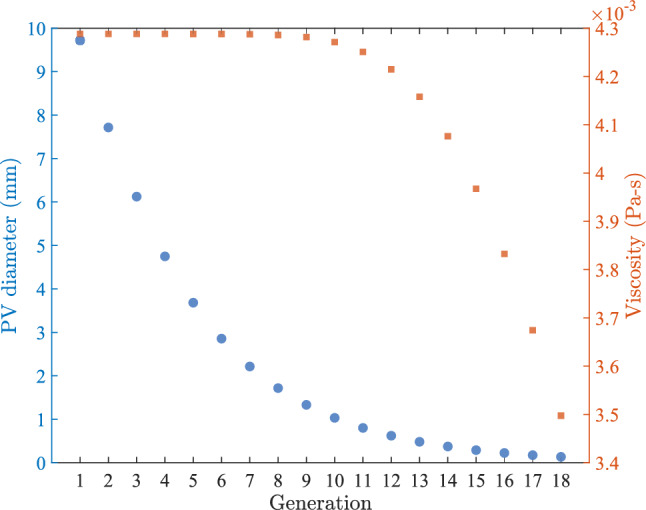
Plot showing how viscosity varies with diameter across generations of branching vasculature. PV diameter as function of branching generation is shown on the left (blue) y-axis and corresponding change in blood viscosity as PV diameter decreases with branching generation is shown on the right (orange) y-axis

We modeled the progression of cirrhosis by reducing the mean conductance of the lumped sinusoid channels and by increasing the heterogeneity of the sinusoids. To vary heterogeneity, the conductance of each of the 30 million lumped sinusoid channels was determined by randomly sampling a log-normal distribution with a user-specified mean $c_{sin}$ and standard deviation $\sigma$. All healthy liver simulations used a mean of $c^0_{sin} = 1.2\times 10^{-5}$ ml/(min$\cdot$mmHg) and a standard deviation normalized with $c^0_{sin}$ of $\sigma /c^0_{sin}=0$, corresponding to a completely homogeneous sinusoidal network with no abnormal fibrosis. We varied the mean conductance of the lumped sinusoid channels from $c_{sin} = 1.2\times 10^{-5}$ ml/(min$\cdot$mmHg) to $1.2\times 10^{-6}$ ml/(min$\cdot$mmHg) across Couinaud segments from early to advance cirrhosis, corresponding to gradual sinusoid constriction. The lower limit of $c_{sin}$ was set at $1.2\times 10^{-6}$ ml/(min$\cdot$mmHg) as tests showed that narrowing the lumped sinusoid channels below this value led to portal vein pressures far beyond the biologically viable range. Increased heterogeneity was implemented by increasing the normalized standard deviation $\sigma /c^0_{sin}$ of the log-normal distribution. The standard deviation $\sigma /c^0_{sin}$ values were varied from 0.035 to 0.16 across segments from early to advanced cirrhosis, where larger standard deviation values correspond to increasing heterogeneity in the sinusoidal network. Note that since exponentiation and multiplicative scaling of a log-normal distribution is still log-normal, one may interpret our model as a log-normal distribution of lumped sinusoid diameter or conductance based on Eq. (1). In Section 2 of the Supplementary Information, we present results analogous to those presented in Sect. 3 below but for Gaussian-distributed lumped sinusoid diameters.

### Diameter and hematocrit dependent viscosity

Our model computes vessel hydraulic conductance using Poiseuille’s law, based on assumptions that the fluid is Newtonian with constant viscosity and that the flow is steady and laminar in a cylindrical tube with a parabolic velocity profile. However, blood exhibits non-Newtonian properties, with viscosity decreasing as shear rate increases, influenced by red blood cell aggregation at low shear rates and cell deformation at higher shear rates. The blood viscosity also increases with increasing hematocrit (i.e., volume fraction of red blood cells in the blood) (Pries et al. 1992). In microvessels, red blood cell size is comparable to vessel diameter, so a continuum description is inadequate and bulk rheology does not directly apply. To incorporate these effects while retaining the Poiseuille form, we replace the constant viscosity in our calculation of conductance with an “apparent viscosity” defined as the viscosity of a Newtonian fluid that would yield the same flow for the same tube geometry and pressure drop. We use the empirical correlation of Pries et al. (1992) to express the relative apparent viscosity ($\eta _{rel}$) as a function of tube diameter and hematocrit, and we compute the apparent viscosity ($\mu _{ap}$) as

6 $$\begin{aligned} \mu _{ap}=\eta _{rel}*\mu _{SM}, \end{aligned}$$

where $\mu _{SM}$ is viscosity of the suspending medium, equal to 0.00134 Pa$\cdot$s based on experimental measurements of human blood plasma (Brust et al. 2013), and $\eta _{rel}$ is written as

7 $$\begin{aligned} \eta _{rel}=1+(\eta _{rel,0.45}-1)\cdot \frac{(1-Hct_D)^C-1}{(1-0.45)^C-1}. \end{aligned}$$

The parameter C describes the curvature of the relationship between relative apparent blood viscosity and hematocrit and is given by

8 $$\begin{aligned} C&=(0.8+e^{-0.075D})\cdot \bigg (-1+\frac{1}{1+10^{-11}\cdot D^{12}}\bigg ) \nonumber \\ &\quad +\frac{1}{1+10^{-11}\cdot D^{12}}. \end{aligned}$$

Finally, $\eta _{rel,0.45}$ is relative viscosity for a hematocrit of 0.45 and is given by

9 $$\begin{aligned} \eta _{rel,0.45}=220\cdot e^{-1.3D}-2.44\cdot e^{-0.06D^{0.645}}+3.2 \end{aligned}$$

The portal vein diameter across branching generations and the corresponding apparent viscosity variation predicted by Eqs. (6–7) are summarized in Fig. 3. Apparent viscosity rises with vessel diameter and approaches an asymptotic value for vessel diameter greater than 1 mm, where microvascular effects are negligible. We set hematocrit to a typical value of 0.45 for human blood at ${37} ^\circ$C. For all lumped sinusoid channels, conductance is computed using an effective viscosity of 0.0018 Pa$\cdot$s defined as the simple average of the apparent viscosities evaluated at the endpoints of the sinusoidal diameter range of 7–15 $\mu$ m (Oda et al. 2003). For simplicity, we do not alter the apparent viscosity of the lumped sinusoids even when their conductance is varied.

## Results

In Sect. 3.1, we first present how sinusoid conductance $c_{sin}$ varies with lumped sinusoid channel diameter and characterize our simulations with different levels of sinusoid heterogeneity $\sigma$ in each Couinaud segment for three separate models of healthy, early, and advanced cirrhotic livers. We quantify volume flow rate and pressure variation in major vessels of HA, PV, RHV, MHV, and LHV in these models in Sect. 3.2. Then, in Sect. 3.3, we describe how perfusion and wall shear stress vary in each segment due to variation in lumped sinusoid conductance (decreased mean conductance and increased heterogeneity). Then in Sect. 3.4, we characterize how portal hypertension changes as the mean sinusoid conductance and sinusoid heterogeneity $\sigma$ increases or decreases. Note that analogous results corresponding to a Gaussian distribution are presented in Section 2 of the Supplementary Information.

### Effects of reducing sinusoid conductance and increasing heterogeneity

Progression of cirrhosis is associated with fibrotic matrix deposition, hepatocyte swelling, and inflammation, all of which may lead to a decrease in the mean sinusoid diameter and increased sinusoid heterogeneity, which we model. A decrease in sinusoid diameter means the sinusoid conductance $c_{sin}$ decreases and from Eq. (1), the rate of change of $c_{sin}$ with diameter can be written as

10 $$\begin{aligned} \begin{aligned} \frac{\textrm{d}c_{sin}}{\textrm{d}(d)}=\frac{4}{d}c_{sin}. \end{aligned} \end{aligned}$$

**Fig. 4:**
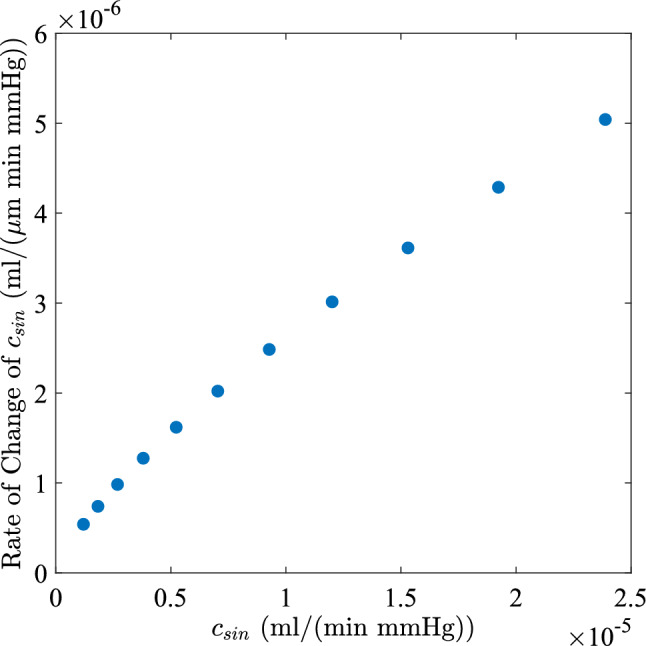
Rate of change of sinusoid conductance with diameter for a single lumped sinusoid channel versus the lumped sinusoid channel conductance. The rate of change is largest for high conductance

The rate of change of sinusoidal conductance $c_{sin}$ with diameter *d* at corresponding $c_{sin}$ is shown in Fig. 4. Here, the rate of change of $c_{sin}$ with *d* increases with increasing $c_{sin}$ or channel size *d*, but this change is nonlinear because the proportionality factor 4/*d* decreases as *d* (or $c_{sin}$) grows. Thus, narrow channels exhibit the smallest conductance increase for a given dilation, while further dilation of wider channels has a more significant effect. In this framework, the healthy case in our model, with $c_{sin} = 1.2 \times 10^{-5}$ ml/(min$\cdot$mmHg), lies in an intermediate regime where diameter changes already have a moderate effect on conductance.

**Table 3:** Mean and standard deviation used to generate the log-normal distributions of the lumped sinusoid network conductance for each Couinaud segment for early and advanced cirrhosis. Note that for the healthy liver, we assume $c_{sin}=c_{sin}^0$ and $\sigma =0$

Couinaud segment		6, 7	5, 8	1, 4a, 4b	2, 3
Early cirrhosis	$c_{sin}/c^0_{sin}$	0.18	0.32	0.5	1
	$\sigma /c^0_{sin}$	0.12	0.08	0.055	0.035
Advanced cirrhosis	$c_{sin}/c^0_{sin}$	0.1	0.16	0.25	0.4
	$\sigma /c^0_{sin}$	0.16	0.13	0.1	0.08

To model the anatomical changes that occur for different stages of cirrhosis, we vary $c_{sin}$ and $\sigma$. As mentioned above, for the healthy liver, we use a uniform value of $c_{sin}=1.2\times 10^{-5}$ ml/(min$\cdot$mmHg) (denoted $c_{sin}^0$) with $\sigma =0$. For different stages of cirrhosis, our model parameters are chosen to reflect the characteristic right-lobe atrophy and left-lobe hypertrophy observed clinically. This modeling approach is reasonable, as studies confirm that fibrosis is often non-uniform and more pronounced in the right lobe (Harbin et al. 1980; Higaki et al. 2023). This pathological finding is translated directly into our model’s parameters: The right lobe is assigned a lower mean sinusoidal conductance ($c_{sin}$) and higher heterogeneity ($\sigma$) to capture atrophy, while the compensating left lobe retains a higher conductance to accommodate the redistributed blood flow. Table 3 lists the values used to set segment-wise sinusoid conductance distributions for early and advanced cirrhosis. For each Couinaud group, we assume a log-normal distribution with mean $c_{sin}$ and standard deviation $\sigma$, presented in a dimensionless form normalized by the healthy reference $c^0_{sin}$. In early cirrhosis, we implement the largest decrease in mean conductance for the right lobe (Segments 6/7 and 5/8), while conductance for the left lobe (Segments 1/4a/4b and 2/3) remains higher. In advanced cirrhosis, mean conductance is further decreased across all segments and $\sigma$ increases.

**Fig. 5:**
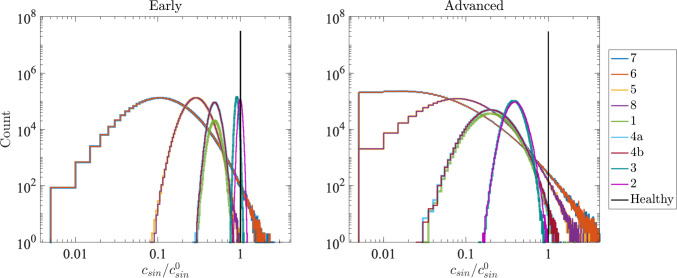
Distribution of lumped sinusoid channel conductance values throughout the sinusoid network for all nine Couinaud segments for livers with (left) early cirrhosis and (right) advanced cirrhosis. Note that some curves corresponding to different Couinaud segments with identical parameters overlap (e.g., 6 and 7); see Table 3

Figure 5 shows distributions of lumped sinusoid conductance for liver network models generated with different log-normal distributions characterized by mean conductance $c_{sin}/c^0_{sin}$ and standard deviation $\sigma /c^0_{sin}$ for early and advanced cirrhosis. The healthy liver case ($\sigma /c^0_{sin}=0$) is indicated in black and corresponds to a single vertical bar at $c_{sin}/c^0_{sin}=1$ on all plots, indicating that all lumped sinusoid channels have the same conductance. For the early cirrhosis model, as the level of heterogeneity increases and the mean conductance decreases (i.e., moving from segments 2/3 in the left liver lobe to segments 6/7 in the right liver lobe), the distributions widen and the peak of the distribution moves to the left. For the advanced cirrhosis model, the shift to the left and broadening of the distribution is strongest, consistent with greater narrowing and variability than in early cirrhosis.

### Alterations in hemodynamics of major vessels for different stages of cirrhosis

**Fig. 6:**
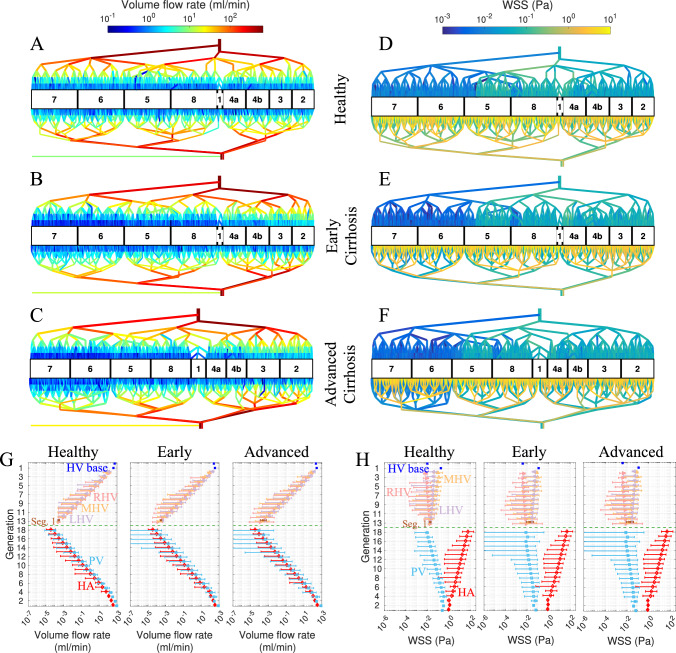
Volume flow rate and wall shear stress throughout the liver. Schematics of whole-liver vasculature showing color encoded (**A**–**C**) volume flow rate and (**D**–**F**) wall shear stress for a healthy (top), early cirrhotic liver (middle), and an advanced cirrhotic liver (bottom). **G**, **H** Plots showing average (symbols) and range (uncertainty bars) for (**G**) volume flow rate and (**H**) wall shear stress in each branching generation with color-coded and labeled vessel segments. In all cases, $c_{col} /c_{liver} = 0.01$. The plots (**A**–**F**) only show the first few branching generations for simplicity

To visualize the hemodynamic impact of cirrhosis, we generated pseudocolor plots of volume flow rate and wall shear stress (WSS) across a few branching generations of the vascular network for three randomly generated livers, each at one of the three stages described above. These plots, shown in Fig. 6A–F, utilize a logarithmic scale to capture changes across generations, and thus comparisons between livers are subtle. Differences in HV flow (top half of each plot) are perhaps most apparent: compared to the healthy case (A), in cirrhosis less flow drains from the right lobe (Segments 7, 6, 5, 8) and more drains from the left lobe (Segments 4a, 4b, 3, 2); this is apparent from the changes in shades of red for the LHV, MHV, and RHV in (B-C) compared to (A). The plots also suggest greater asymmetry in the left versus right-lobe volume flow rates for early and advanced cirrhosis, since (B-C) show some color differences (e.g., there is more dark blue near segments 6 and 7 compared to 2 and 3). Plots of WSS (D-F) confirm that WSS is highest for the HA and lower throughout the PV and HV, as expected. The WSS in the PV decreases from healthy conditions to early cirrhosis and is lowest in advanced disease, consistent with higher intrahepatic resistance for a given inflow. On the HV side, WSS trends depend on the segment setup; segments with higher $c_{sin}/c^0_{sin}$ and modest $\sigma /c^0_{sin}$ (Segments 1, 4a/4b, 3, 2) tend to maintain or show a slight increase in WSS, while narrowed, more heterogeneous right-lobe segments (7, 6, 5, 8) show lower WSS. This pattern reflects both the flow shift toward less resistive territories and the local diameter/conductance changes.

The volume flow rate and WSS (mean and range) across all branching generations in different vessels, comparing the healthy case to the progression of cirrhosis, are shown in Fig. 6G–H. The plots characterize the flow rate and WSS variability (represented by the uncertainty bars) between parallel vessels at a given generation even in the healthy case. Across different cirrhosis stages, the volume flow rate and WSS remain almost indiscernible on a logarithmic scale for the HA. However, mean values for both volume flow rate and WSS are shifted slightly higher for the LHV and slightly lower for the RHV in early/advanced cirrhosis, compared to the healthy liver; additionally, the HV variability is generally larger for early/advanced cirrhosis, especially in the vicinity of the sinusoids (generations 10–13). Perhaps the most visible difference in early/advanced cirrhosis compared to the healthy liver is the significant increase in variability of volume flow rate and WSS, especially for higher generations in which the uncertainty bars span upward of four orders of magnitude. This widening of the range is driven by the segmental sinusoid heterogeneity. As cirrhosis progresses, the narrowest lumped sinusoid channels (in the most heterogenous segments) receive significantly less blood flow, which extends the data range of volume flow rate and WSS toward much lower values. While the less heterogeneous segments receive a compensatory higher flow, the overall expansion of the range is dominated by this large drop in flow rate and WSS to the narrowed and more heterogeneous segments.

**Fig. 7:**
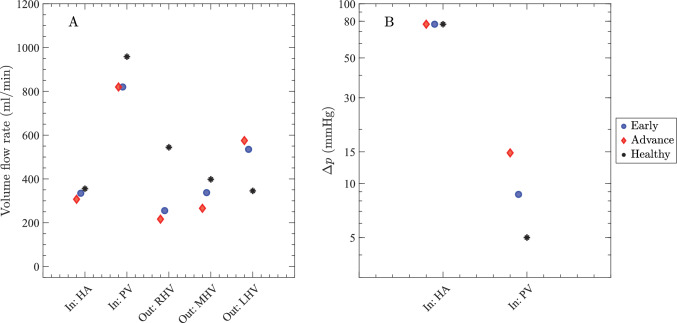
Plots of volume flow rate and inlet pressure differences (relative to the vena cava) in the liver. **A** Volume flow rate through major vessels of HA, PV, RHV, MHV & LHV, and **B** inlet HA and PV pressure differences for an early cirrhotic liver (blue circles), an advanced cirrhotic liver (red diamonds), and a healthy liver (black asterisks). In all cases, $c_{col} /c_{liver} = 0.01$

The volume flow rate through the liver, as well as the inlet HA and PV pressure differences for different cirrhosis stages, are plotted in Fig. 7. With the fixed portal vein inflow boundary condition, the PV flow rate remains the same in cases of early and advanced cirrhosis, which is about 15% lower than in the healthy case. The hepatic artery flow declines slightly as cirrhosis progresses due to the decrease in sinusoid conductance (and fixed hepatic vein pressure). Hepatic vein outflows redistribute so that RHV drainage falls the most, MHV drainage shows a smaller drop, and LHV drainage increases, consistent with right-lobe atrophy and left-lobe hypertrophy. These changes are most pronounced in the advanced stage. The PV inlet pressure difference rises with severity, from about 5 mmHg in healthy conditions to about 8 mmHg in early cirrhosis, then about 15 mmHg in advanced disease. The advanced value is within the portal hypertension range (Abraldes et al. 2014; Bochnakova 2021) and reflects higher intrahepatic resistance. Since the network generation involves random sampling of splitting numbers from a normal distribution, we also performed multiple realizations with fixed input parameters to quantify sampling variability. The results shown in Fig. 7 remained high robustness: The large number of segments effectively dampens these local fluctuations, resulting in volume flow rate and pressure variations of less than 1% across realizations.

**Fig. 8:**
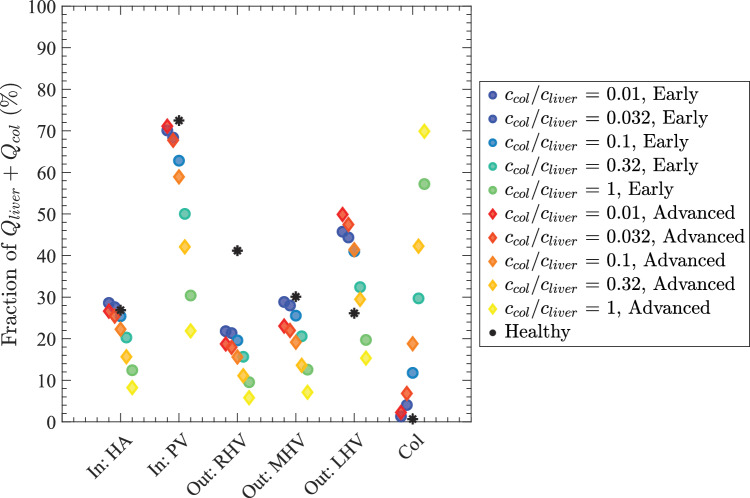
Fraction of total volume flow rate through the liver $Q_{liver}$ and the collateral $Q_{col}$ in major vessels of HA, PV, RHV, MHV & LHV plotted for varying $c_{col}$ for a healthy liver (black asterisks), a early cirrhotic liver (blue–cyan circles), and an advanced cirrhotic liver (red–yellow diamonds). Note that for a given marker, HA, PV, and Col sum to 100%, and RHV, MHV, LHV, and Col sum to 100%

Next, we discuss how collateral dilatation affects the volume flow rates. Collateral dilatation leads to a strong increase in collateral conductance $c_{col}$ (due to the $d^4$ dependence in Eq. (1)). We report $c_{col}$ as a fraction of total liver conductance ($c_{liver} = 176$ ml/[min$\cdot$mmHg]) for a straightforward interpretation of how much blood is shunted through collateral routes (the liver and collaterals may be approximated as parallel blood flow pathways, so when $c_{col}=c_{liver}$, blood flow is distributed approximately evenly between the two routes). Figure 8 shows how flow fractions are partitioned among inflow (HA, PV), outflow through the hepatic veins (RHV, MHV, LHV), and the collateral pathway across five collateral conductance values: $c_{col}/c_{liver}=0.01,\,0.032,\,0.10,\,0.32,\,1.0$ for healthy, early, and advanced cirrhosis. In the healthy liver at $c_{col}/c_{liver}=0.01$, the plotted fractions are HA = 27%, PV = 73%, RHV = 41%, MHV = 30%, LHV = 26%, and collateral = 0.67%. With low collateral conductance ($c_{col}/c_{liver}\le 0.032$), nearly all inflow exits via the hepatic veins in all three cases. As $c_{col}/c_{liver}$ increases, an increasing share of the total $Q_{liver}+Q_{col}$ is diverted through collaterals, adding a parallel pathway that effectively lowers overall hydraulic resistance in early and advanced cirrhosis. In Fig. 8, this appears as decreasing fractions for HA, PV, and all three HVs as $c_{col}/c_{liver}$ rises (blue $\rightarrow$ cyan for early and red $\rightarrow$ yellow for advanced). Compared to the healthy liver, the redistribution across hepatic veins is not uniform: The RHV loses the largest share, the MHV decreases less, and the LVH increases up to $c_{col}/c_{liver}\le 0.32$ before declining at higher values, consistent with right-lobe atrophy and left-lobe hypertrophy. In advanced cirrhosis, where intrahepatic resistance and portal pressure are higher, the shift to collateral drainage occurs at lower $c_{col}/c_{liver}$ and the fractional change is larger. When $c_{col}/c_{liver}$ is very large ($\approx 1$), about 70% of the total flow is routed through collaterals and only $\sim 30\%$ passes through the liver, so the HV portions decrease accordingly while the HA and PV fractions of the total decrease in proportion to the growing collateral component.

### Couinaud segment hemodynamics

**Fig. 9:**
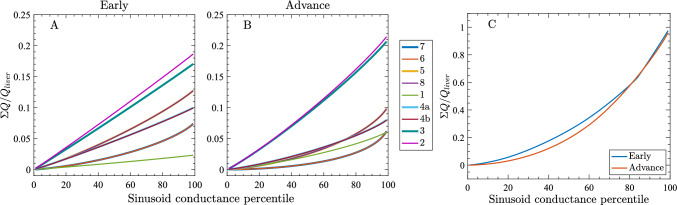
Plots of total flow fraction versus lumped sinusoid channel conductance percentile. Percentiles indicate all lumped sinusoid channels at or below a given diameter (for example, the 50th percentile includes the smallest half of all lumped sinusoid channels and the 100th percentile includes all lumped sinusoid channels). The total flow fraction $\Sigma Q/Q_{liver}$ is defined as the sum of the volume flow rate through all lumped sinusoid channels at a given percentile, divided by the total volume flow rate through the liver. Plots include segment-wise distribution for **A** early cirrhotic liver and **B** advanced cirrhotic liver, as well as **C** distribution through the entire liver. Note that some curves in panel **A** or **B** overlap (4a & 4b, 5 & 8, 6 & 7)

We next describe how sinusoid narrowing and increased heterogeneity across Couinaud segments affect volume flow rate and liver perfusion. For the early and advanced cirrhosis models, we varied $c_{sin}/c^0_{sin}$ and $\sigma /c^0_{sin}$ by segment, sorted the lumped sinusoid conductances in ascending order segment-wise and for the whole liver, and then computed the cumulative fraction of blood flow through all vessels up to a given conductance (expressed as a conductance percentile). Figure 9 plots the cumulative flow fraction, normalized by total liver flow, as a function of conductance percentile for each segment from early cirrhosis (Fig. 9A) to advanced cirrhosis (Fig. 9B), as well as for the entire liver (Fig. 9C).

When heterogeneity in a segment is minimal ($\sigma /c^0_{sin}<0.1$ for segments 1–4), the constant or near-constant vessel conductance leads to minimal variations in volume flow rate through the lumped sinusoid channels. This results in a linear relationship between the cumulative volume flow rate and lumped sinusoid channel conductance percentile, indicating more uniform distribution of flow across the sinusoids, consistent with higher $c_{sin}/c^0_{sin}$ and smaller $\sigma /c^0_{sin}$. As $\sigma /c^0_{sin}$ increases and $c_{sin}/c^0_{sin}$ decreases, low-conductance sinusoids contribute progressively less to total flow, while a small set of high-conductance sinusoids carries disproportionately larger amounts of blood flow, yielding curves that remain flat over much of the range and then rise steeply at large percentile values. In the early cirrhosis model, right-lobe segments (5-8) with reduced $c_{sin}/c^0_{sin}$ and larger $\sigma /c^0_{sin}$ show steeper rises at higher sinusoid percentile, indicating flow concentrated in a few higher-conductance sinusoids. In advanced cirrhosis, these contrasts intensify: Right-lobe curves shift further toward higher sinusoid percentiles (even more flow through the high-conductance channels), whereas left-lobe curves move toward earlier accumulation with more even distribution, reflecting the segment-wise mean and standard deviation settings in the model. Figure 9C shows the flow fraction as a function of the whole-liver conductance percentile, irrespective of the segment-wise distribution. The early (blue) and advanced (orange) cirrhosis curves are similar, but the early curve lies above the advanced curve across most of the range. This fairly small offset indicates a more even distribution of flow across sinusoids in early cirrhosis, while advanced cirrhosis shifts a larger proportion of flow toward the highest-conductance lumped sinusoid channels.

**Fig. 10:**
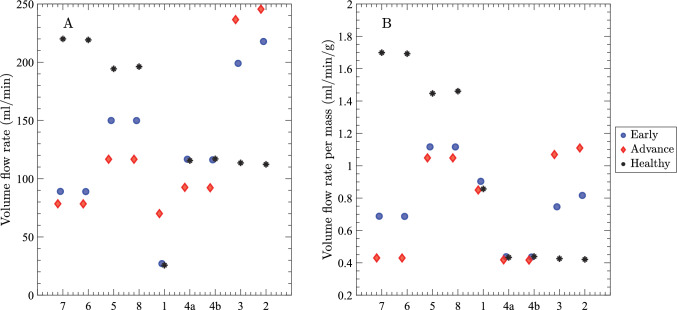
Plots of volume flow rate and perfusion in the liver for different Couinaud segments. **A** Volume flow rate, and **B** perfusion in individual Couinaud segments for an early cirrhotic liver (blue circles), an advanced cirrhotic liver (red diamonds), and a healthy liver (black asterisks). In all cases, $c_{col} /c_{liver} = 0.01$

Next, we summarize segmental volume flow rate (ml/min) and perfusion (ml/(min$\cdot$g)) for the three cases, as shown in Fig. 10. In the right-lobe segments (7, 6, 5, 8), volume flow rate decreases comparing the healthy liver to early cirrhosis, and a further decline occurs in advanced cirrhosis (Fig. 10A). In contrast, the left lateral segments (3, 2) show higher flow in early cirrhosis (compared to the healthy liver), which is even further elevated in advanced cirrhosis. The perfusion panel mirrors these trends: the right-lobe perfusion decreases further with early than advanced cirrhosis, whereas the left lateral segments progressively increase (Fig. 10B). These patterns reflect the segmental conductance changes presented in Table 3: lower mean conductance and higher heterogeneity in the right lobe populate that lobe with a large number of very low-conductance channels, which diverts flow away from those segments. While higher mean conductance and lower heterogeneity in the left lobe produces a distribution more tightly concentrated around higher conductance, drawing flow toward those segments. With total portal inflow fixed, the network preferentially routes blood toward higher-conductance channels, accentuating left-right differences with cirrhosis progression.

**Fig. 11:**
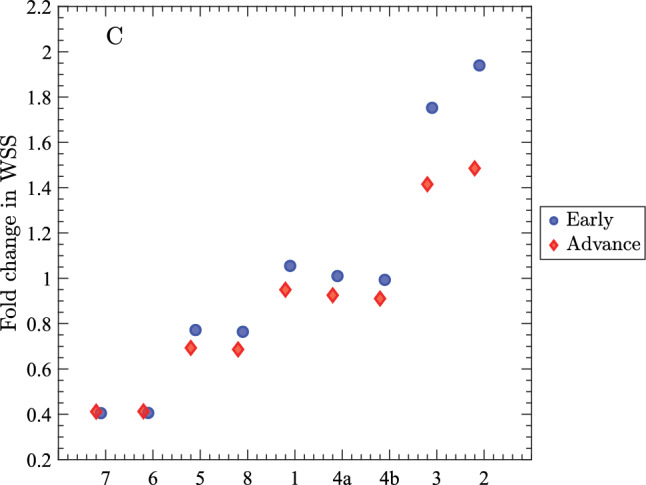
Plots of mean fold change in WSS for an early cirrhotic liver (blue circles), an advanced cirrhotic liver (red diamonds), and a healthy liver (black asterisks). In all cases, $c_{col} /c_{liver} = 0.01$

We also report segment-wise changes in mean wall shear stress (WSS), relative to the healthy liver, for the lumped sinusoid channels for early and advanced cirrhosis (Fig. 11). In the right-lobe segments (7, 6, 5, 8), WSS decreases (WSS fold change $<1$) from healthy conditions to early cirrhosis, and this falls further in advanced disease, consistent with the drop in segmental volume flow rate and rerouting of flow toward the left lobe. In the left-lobe segments (1, 4a/4b), WSS is maintained or modestly elevated, with slightly lower values in the advanced case. In the inferior-left group (2, 3), WSS increases (WSS fold change $>1$), with the largest rise in early cirrhosis. These trends reflect two competing effects: WSS rises with higher volume flow rate and smaller diameter or conductance, but declines when flow is reduced. In the right lobe, the flow reduction dominates, so WSS tends to decline even with sinusoids narrowing. In the left lobe, higher sinusoid conductance allows a higher flow rate for a given pressure drop; this increase is sufficient to counter the WSS decrease expected from diameter enlargement (or increased conductance), yielding WSS that is comparable to or slightly above healthy values. Overall, progression from early to advanced cirrhosis amplifies the left–right contrast.

### Changes in portal pressure due to alterations in sinusoid conductance

**Fig. 12:**
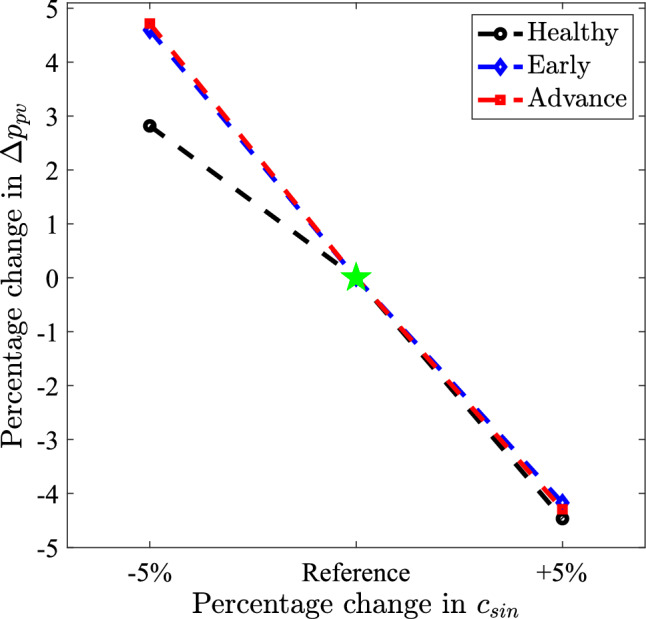
Characterization of how portal vein pressure difference $\Delta p_{pv}$ varies with liver-wide changes in sinusoid conductance $c_{sin}$. Plots show percentage change in $\Delta p_{pv}$ for varying all $c_{sin}$ by $\pm 5\%$. In all cases, $c_{col}/c_{liver}=0.01$. Note we do not show the pressure sensitivity due to change in $\sigma$ because varying $\sigma /c^0_{sin}$ by $\pm 5\%$ with fixed mean $c_{sin}/c^0_{sin}$ lead to $< 1\%$ change in $\Delta p_{pv}$

To quantify how the portal vein pressure difference ($\Delta p_{pv}$) changes due to alterations in lumped sinusoid conductance, we varied each sinusoid conductance $c_{sin}$ by $\pm 5\%$, as plotted in Fig. 12. In the healthy case ($\sigma =0$), varying $c_{sin}$ has a substantially nonlinear, asymmetric effect on $\Delta p_{pv}$: a $5\%$ decrease in all $c_{sin}$ increases $\Delta p_{pv}$ by only $2.8\%$, whereas a $5\%$ increase reduces $\Delta p_{pv}$ by $4.5\%$ (black points in Fig. 12). This asymmetry arises because of the nonlinear rate of change of sinusoid conductance with diameter at a given $c_{sin}$ (Fig. 4). Increasing $c_{sin}$ shifts the system into a regime of higher sensitivity where enlarging the sinusoids produces a larger increase in overall conductance; hence, a significantly lower $\Delta p_{pv}$ is required to drive the fixed PV flow. However, decreasing all $c_{sin}$ leads to a smaller reduction in overall conductance and, thus, a smaller increase in $\Delta p_{pv}$.

The early and advanced cirrhosis cases are more sensitive to decreases in $c_{sin}$ compared to the healthy case, showing a 4.7% increase in $\Delta p_{pv}$ with a 5% decrease in conductance (Fig. 12, blue and red points). However, increasing $c_{sin}$ by $5\%$ results in a similar drop in $\Delta p_{pv}$ (4.3%) across both cases. This behavior arises from the log-normal distribution in cirrhosis cases (Fig. 5). Most lumped sinusoids fall in a low-conductance, low-sensitivity regime where any changes in these vessels have a small effect on overall conductance (Fig. 4). However, due to the wide distribution, a high-conductance tail ($c_{sin}/c^0_{sin}>1$) persists even when $c_{sin}$ is decreased. Because the low-conductance vessels contribute minimally to flow, the system’s overall sensitivity is dominated by this subset of higher-conductance channels (illustrated in Fig. 9). Changing $c_{sin}$ for this subset has a large influence on the effective intrahepatic conductance, producing an almost proportionate effect on $\Delta p_{pv}$. In summary, the healthy liver demonstrates moderate hemodynamic stability to reductions in mean sinusoid conductance; however, for cirrhotic livers, a disproportionate fraction of flow passes through the highest-conductance vessels, leading to a greater elevation in portal pressure due to reductions in $c_{sin}$.

On the other hand, varying segment-wise $\sigma /c^0_{sin}$ by $\pm 5\%$ with fixed mean $c_{sin}/c^0_{sin}$ leads to $<1\%$ change in $\Delta p_{pv}$, indicating that changes in $\sigma /c^0_{sin}$ have only a minor impact on portal pressure. Altering $\sigma /c^0_{sin}$ for our log-normal distributions primarily reshapes the distribution of the low-conductance vessels, and although this also modifies the density of high-conductance channels, the magnitude of this shift is insufficient to significantly alter the effective intrahepatic conductance, so $\Delta p_{pv}$ remains nearly unchanged.

## Discussion and conclusions

This manuscript presents a numerical model of blood flow through the human liver in which we have investigated hemodynamic alterations that occur in different stages of cirrhosis. Clinically, worsening cirrhosis is associated with increasing fibrosis and elevated intrahepatic resistance which elevates portal pressure, leading to the formation of extrahepatic collaterals (varices, splenorenal shunts, etc.) that divert some portal flow away from the liver. We numerically investigated this phenomenon by decreasing lumped sinusoid conductance, increasing lumped sinusoid heterogeneity, and dilating a collateral vessel. Our model uses a lumped parameter approach which enables realistic simulation of blood flow through tens of millions of vessel segments, constructed based on statistics of human hepatic vasculature (Table 1), in a matter of minutes. An important aspect of this study is the modification made to the linear algebra, given by Eq. (5), which allowed for different inlet boundary conditions: fixed pressure for the hepatic artery, but fixed volume flow rate for the portal vein. This is arguably a more biologically accurate boundary condition, as all blood flow from the gut must drain to the portal vein (or collaterals) since this vasculature does not possess any mechanism for regulating blood flow in response to increased hepatic resistance. Implementation of this improved boundary condition enabled a straightforward approach for evaluating changes in $\Delta p_{pv}$ as intrahepatic conductance varied. We also significantly advanced the accuracy of this model by implementing an “apparent viscosity” that accounts for non-Newtonian properties of blood as a function of vessel diameter (assuming a hematocrit value of 0.45). Insights obtained from this model are summarized in the following subsections.

### Effects of varying lumped sinusoid conductance

In this study, we tested the effects of varying the segment-wise lumped sinusoid conductance $c_{sin}$ (by decreasing the mean $c_{sin}/c^0_{sin}$ and increasing the heterogeneity parameter $\sigma /c^0_{sin}$ for early and advanced cirrhosis). This was motivated by our histological images (Fig. 2) and reports in the literature (Rockey and Chung 1998; Greuter and Shah 2016; Yoon et al. 2013) indicating that cirrhosis is associated with alterations in microvascular architecture, hepatocyte swelling, irregular deposition of collagen, inflammation, and neovascularization. We first characterized the effects of varying $c_{sin}/c^0_{sin}$ for a single lumped sinusoid channel. We found that the rate of change in conductance is greater at higher $c_{sin}/c^0_{sin}$ values (Fig. 4), suggesting that increased heterogeneity in the lumped sinusoid channels will alter organ-level flows due to the disproportionately strong influence of lumped sinusoid channels with large $c_{sin}$. For early and advanced cirrhosis, the total portal flow remained fixed since we imposed a fixed volume flow rate boundary condition. However, the decreased mean hydraulic conductance and increased heterogeneity of the lumped sinusoid channels reduced the overall conductance, leading to moderate elevation of the portal pressure (Fig. 7B), consistent with clinical measurements. Segments with lower mean conductance (such as segments 6 and 7; see Table 3) exhibited reduced flow (Fig. 10), despite having the largest heterogeneity $\sigma$. In contrast, segments with higher mean conductance (such as segments 2 and 3) received an increased fraction of the overall flow. The model predicts substantial decreases in right-lobe WSS (segments 6 and 7 and to a lesser extent 5 and 8) and increases in the left-lobe WSS (segments 2 and 3), as shown in Fig. 11.

An important prediction from our simulations is that liver segments with greater levels of sinusoid heterogeneity (i.e., large $\sigma /c^0_{sin}$) will exhibit an increasingly disproportionate fraction of the blood flow passing through the few sinusoids with the largest conductance (Fig. 9A-B). This observation identifies a likely major contributing factor to liver dysfunction, as heterogeneous flow will hamper the vital blood filtration functions of the liver, which occur at the sinusoids. This hypothesis is supported by prior modeling studies which have investigated heterogeneous liver perfusion and concluded that it likely impairs drug metabolism (Schwen et al. 2014, 2015).

### Hemodynamic alterations in major vessels

The major-vessel results indicate that the total inflow to the liver (excluding collateral flow) remains similar across healthy and cirrhotic conditions, with small decreases in inflow for both hepatic artery and portal vein in cirrhosis (Fig. 7A); the former is a consequence of decreased intrahepatic conductance, while the latter ($\sim$15% portal flow reduction) was prescribed using a fixed volume flow rate boundary condition based on clinical measurements (Brunsing et al. 2021). Plots of the volume flow rate across branching generations (Fig. 6G) show increased variability in the portal flow for the few branching generations just before the sinusoids, in both early and advanced cirrhosis, suggesting that increased sinusoidal heterogeneity also alters microvascular portal flow (but variability of hepatic artery flow is minimally altered). Similar changes in WSS of the microvascular portal flow are even more substantial (Fig. 6H).

On the outflow side, the right hepatic vein drainage (associated with segments 7, 6, 5, 8) decreases substantially while the left hepatic vein drainage (associated with segments 4a, 4b, 3, and 2) increases substantially (Fig. 7A). The drainage through the middle hepatic vein (associated with segments 4a, 4b, 1, 5, and 8) only decreases a small amount. These results follow from the assigned segmental means and standard deviations of sinusoid conductance (Table 3), wherein we modeled cirrhosis by enforcing the greatest reduction in mean conductance in the right liver. Some increase in variability of volume flow rate and WSS in the hepatic vein is also apparent, especially for the last few generations in the vicinity of the sinusoids (Fig. 6G–H).

We assumed hepatic artery pressure $\Delta p_{ha}$ remains constant for all 3 cases (prescribed by a fixed pressure boundary condition). However, portal venous pressure $\Delta p_{pv}$ rises as sinusoid conductance falls (Fig. 7B), and the rise is dominated by the drop in the mean sinusoidal conductance $c_{sin}/c^0_{sin}$ (Fig. 12). In our simulations, $\Delta p_{pv}$ progresses from 5 mmHg (healthy) to 8 mmHg (early cirrhosis) to 15 mmHg (advanced cirrhosis), consistent with clinical reports of portal hypertension in advanced disease (Abraldes et al. 2014; Bochnakova 2021). Since portal vasculature does not possess any mechanism for regulating blood flow in response to increased hepatic resistance, collateral vessels provide an alternate path that offloads a growing proportion of total portal flow as $c_{col}/c_{liver}$ increases. We varied $c_{col}/c_{liver}$ from 0.01 to 1.0 and characterized how the total collateral flow $Q_{col}$ grows as a fraction of total flow $Q_{liver}+Q_{col}$ (Fig. 8). Our results indicate that for very large collateral conductance ($c_{col}/c_{liver} =1$), about 70% of the total flow bypasses the liver (with the remaining $\sim 30\%$ flowing through the liver). This prediction is supported by clinical measurements which report a “shunt index” (a rough estimate of the fraction of blood flow that bypasses the liver) as high as 89%, indicating a large fraction of total blood flow does indeed bypass the liver in advanced cirrhosis cases (Kashiwagi et al. 1988; Fukui et al. 1993). Since portal vein flow was held constant in our model, the case with $c_{col}/c_{liver} = 1$ constitutes a major increase in overall (liver plus collateral) blood flow, equal to about 3 times the total volume flow rate in the healthy liver. This is generally consistent with several established features of advanced cirrhosis, including increased cardiac output, splanchnic vasodilation, and hyperdynamic circulation (Kim et al. 2010).

### Alterations in hemodynamics of Couinaud segments

Segmental changes in volume flow rates (Fig. 10A), perfusion (Fig. 10B), and WSS (Fig. 11) follow from our imposed segment-wise changes in conductance for the healthy and cirrhotic liver. Indeed, for the parameters we implemented (Tables 1, 2 and 3), the net blood flow through the cirrhotic liver redistributed among the Couinaud segments, capturing the right-lobe atrophy and left-lobe hypertrophy reported in the literature (Harbin et al. 1980; Higaki et al. 2023). Right-lobe segments 7, 6, 5, and 8 show the largest reductions in volume flow rate, perfusion, and WSS from healthy to early cirrhosis, to advanced cirrhosis. Segments 1, 4a, and 4b are relatively unchanged, while for segments 2 and 3 there is substantial elevation in flow and WSS. These alterations in flow and WSS provide a likely mechanism driving right-lobe atrophy and left-lobe hypertrophy due to reduction/enhancement of delivery of hepatotrophic factors and endothelial signaling that promotes vascular remodeling. Higaki et al. (2023) recently showed that the extent of liver atrophy-hypertrophy is closely associated with increased (and decreased) left (and right) portal flow.

### Portal hypertension

Our sensitivity analysis tested the effects of changing lumped sinusoid conductance $c_{sin}$ (Fig. 12) and concluded that changes in mean conductance is more important than changes in heterogeneity (i.e., $\sigma /c^0_{sin}$) with regard to changes in portal pressure. Reductions in the mean value of $c_{sin}/c^0_{sin}$ and increases in $\sigma /c^0_{sin}$ for each Couinaud segment for early and advanced cirrhosis (Table 3) are intended to model fibrotic matrix deposition, distortion of the microvascular architecture, hepatocyte swelling, inflammation, and neovascularization, much of which is evident in our histological images in Fig. 2. The true progression of cirrhosis is even more complex, as cirrhotic portal hypertension is associated with a reduction in nitric oxide production (a strong vasodilator) and increased release of vasoconstrictive factors (Li et al. 2025). Nonetheless, our model’s prediction that increased heterogeneity has a minor effect on portal hypertension is generally supported by clinical literature. Since increased heterogeneity has a tendency to *reduce* portal pressure (via disproportionately increased flow through the lumped sinusoids with the highest conductance), it provides a mechanism that competes with collateral dilatation to oppose portal hypertension. Since collateral dilatation is essentially ubiquitous in advanced cirrhosis (although the details of which channels enlarge and to what extent are highly variable) (Sharma and Rameshbabu 2012), increased sinusoid heterogeneity is very likely a small or negligible mechanism for reduction of portal hypertension.

### Limitations and future extensions

Simulations presented in this manuscript offer mechanistic insights into complex hemodynamical and anatomical changes that occur for different stages of cirrhosis, including alterations to volume flow rates and pressures. This modeling provides fundamental insights into liver pathology and helps explain clinical observations, such as mechanisms contributing to right liver atrophy and left liver hypertrophy. This study is also an important component of our long-term goals of developing computationally efficient patient-specific simulations that improve outcomes in living donor liver transplantation. For example, future patient-specific modeling of both the donor and recipient may be leveraged to obtain numerical predictions of donor liver conductance and recipient collateral conductance. These quantities can then be used to predict the post-operative proportion of blood that will be shunted through collaterals, informing clinicians of the likelihood of liver regeneration in the patient and the potential need for collateral ligation.

Several important limitations to the numerical models presented herein should be noted, and future extensions to this work should improve the accuracy of the model’s simplifying assumptions. First, our model assumes each portal triad is connected to each adjacent centrilobular (hepatic) vein via a single lumped sinusoid channel (Fig. 1H–I) which captures the equivalent conductance of the complex network of sinusoid vessels. Imaging similar to that reported by Ishikawa et al. (2021) could be used to more accurately model the details of the sinusoid network, although this would substantially increase the computational cost of the simulations. Since the sinusoids are a complex network of branching and merging channels, this extension may reveal more nuanced and complex effects arising due to growing sinusoid heterogeneity. Importantly, no data exist (to the best of our knowledge) for determining whether a log-normal distribution is an appropriate choice for modeling sinusoidal alterations due to cirrhosis and what values of $c_{sin}$ and $\sigma$ (Table 3) are most accurate for the healthy and cirrhotic liver. Our approach to modeling collateral vessels could be substantially improved. In our current approach, we model only a single lumped collateral vessel (Fig. 1F) which is assumed to be perfectly in parallel with the liver. Extending the model anatomy to include the upstream anatomical details of the portal vein (incorporating the splenic vein, superior mesenteric vein, etc.) would facilitate more accurate modeling of collaterals. In addition, we assume that collateral dilatation has no upper limit, but spontaneous hemoperitoneum caused by rupture of the umbilical vein collateral has been reported in end-stage liver cirrhosis (Honmyo et al. 2021). Our modeling could be extended to indicate a volume flow rate and/or pressure at which rupture would occur. Collateral angiogenesis (formation of new blood vessels) is another biological phenomenon not captured by our model that would likely be challenging but feasible to incorporate. Finally, our model does not capture the true, dynamic changes in blood pressures and volume flow rates, such as those that occur following meals (Dauzat et al. 1994; Roldán-Alzate et al. 2015) or as a consequence of the hepatic artery buffer response (Gülberg et al. 2002; Eipel et al. 2010).

In addition to the possible model extensions listed above, these simulations could be adapted to capture hepatic blood flow in an animal model, which could help validate the numerical predictions presented here. Use of an animal model would facilitate in vivo measurement aimed at quantifying anatomical and hemodynamic alterations that occur at intermediate stages of liver disease. Such highly invasive surgeries and in vivo imaging are not feasible in humans. In addition, environmental factors in animal studies can be highly controlled, in contrast to patients. Of course, differences in animal versus human liver pathology would need to be considered (Delire et al. 2015), and results may vary depending on the method employed to impose liver disease (Nevzorova et al. 2020).

## Supplementary Information

Below is the link to the electronic supplementary material.

Supplementary file 1 (pdf 475 KB)

## Data Availability

The simulation data that support the findings of this study are available from the corresponding authors upon reasonable request.
