# Supplementary material for: Lumped parameter modeling of changes in liver hemodynamics due to cirrhosis
Source: Biomech Model Mechanobiol. 2026 May 22;25(3):47. doi: 10.1007/s10237-026-02055-9 (PMC13197283; doi:10.1007/s10237-026-02055-9)
Supplement: Supplementary file 1 — (pdf 475 KB) [file 10237_2026_2055_MOESM1_ESM.pdf]

# Supplementary Information for

## Lumped parameter modeling of changes in liver hemodynamics due to cirrhosis


Edith Luveina Joseph[1†], Himanshi Saini[1†], Usha Kini[2], Timothy L. Pruett[3], Joseph Sushil Rao[3,4*], Jeffrey Tithof[1*]

[1]Department of Mechanical Engineering, University of Minnesota, 111 Church St SE, Minneapolis, 55455, Minnesota, US.
[2]Department of Pathology, St. John's National Academy of Health Sciences, Bangalore, 560034, Karnataka, India.
[3]Division of Solid Organ Transplantation, Department of Surgery, University of Minnesota, Minneapolis, 55455, Minnesota, US.
[4]Schulze Diabetes Institute, Department of Surgery, University of Minnesota, 420 Delaware St SE, Minneapolis, 55455, Minnesota, US.

*Corresponding author(s). E-mail(s): jrao@umn.edu; tithof@umn.edu;
†These authors contributed equally to this work.


## 1 Lumped Parameter Hydraulic Network Mathematical Formulation

We assume fluid flows through a uniform pipe of diameter $d$. The volume flow rate $Q$ of the fluid is then related to the pressure drop $\Delta p$ across the pipe as:

$$\Delta p = \frac{128\mu L Q}{\pi d^4} \tag{S1}$$

where $\mu$ is the dynamic viscosity and $L$ is the length of the pipe. Equation (S1) is referred to as the Hagen-Poiseuille equation. Now suppose we denote $R = \frac{128\mu L}{\pi r^4}$. Then we can write:

$$\Delta p = QR \tag{S2}$$

where $R$ is referred to as the "hydraulic resistance." It is informative to note that this equation is analogous to Ohm's law for an electrical circuit, given by $\Delta V = I\mathcal{R}$,

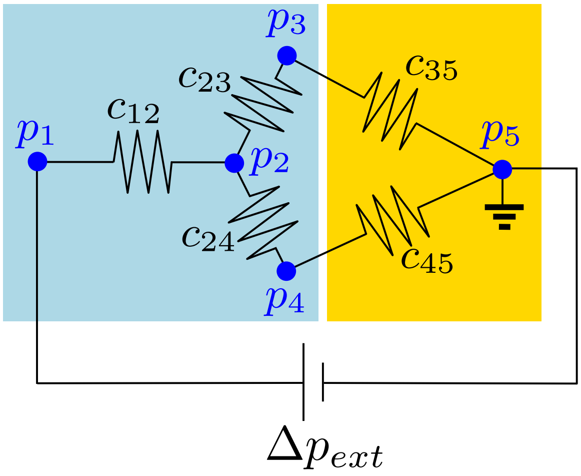


**Fig. S1** A small and simple hydraulic network model.

where $\Delta V$ is the voltage drop, $I$ is the current, and $\mathcal{R}$ is the electrical resistance. Engineers and scientists often use Ohm's law to estimate current and voltage drops in complex circuits. We can analogously use equation (S2) to model fluid flow through complex branching fluid flow networks.

We start with the relatively simple branching network shown in Figure S1 which then can be extended to more complex networks as presented in the main text. In this figure, flow is driven through the network by a single pressure source labeled "$\Delta p_{\text{ext}}$" (analogous to a battery). There are five nodes at which a value of the pressure is defined ($p_1$ to $p_5$), and the last one has a symbol indicating that it is *grounded* (i.e., $p_5 = 0$), which is again analogous to an electrical circuit. Each node is connected to another node with a resistor in between, and the resistors are labeled $c_{ij}$ where $i$ is the *upstream* node and $j$ is the *downstream* node (note that current flows clockwise, from $p_1$ to $p_2$ to $p_3/p_4$, etc). These $c_{ij}$ values are known as the *conductance*, which is the reciprocal of hydraulic resistance:

$$c_{ij} = 1/R_{ij} \tag{S3}$$

where $R_{ij}$ is the resistance connecting nodes $i$ and $j$. Hence, we can rewrite equation (S2) as:

$$c_{ij}(\Delta p)_{ij} = Q_{ij} \tag{S4}$$

where $c_{ij}$ is the conductance of the resistor connecting nodes $i$ and $j$, $(\Delta p)_{ij} = p_i - p_j$ is the pressure drop across the resistor, and $Q_{ij}$ is the volume flow rate through the resistor.

To mathematically describe the network shown in Figure S1, we formulate a linear algebra problem based on Kirchhoff's first law: "The algebraic sum of currents in a network of conductors meeting at a point is zero." For a fluid flow, this law is equivalent

to the continuity equation (i.e., conservation of mass). This law simply states that at any given node, the total amount of fluid flowing in has to match the total amount of fluid flowing out. We can write this mathematically as:

$$\sum_{k=1}^{n} Q_k = 0 \tag{S5}$$

where the summation is applied over all $n$ branches connected to a given node. For example, at node 3 (labeled "$p_3$" in Figure S1), the inflow is given by:

$$Q_{23} = -c_{23}(p_2 - p_3) \tag{S6}$$

and the outflow is given by:

$$Q_{35} = c_{35}(p_3 - p_5). \tag{S7}$$

Note that $p_2 > p_3 > p_5$ so overall $Q_{23} < 0$ and $Q_{35} > 0$. In other words, we have chosen the convention that the volume flow rate *into a node* is *negative* and the volume flow rate *out of a node* is *positive*. To satisfy continuity, we must have:

$$Q_{23} + Q_{35} = -c_{23}(p_2 - p_3) + c_{35}(p_3 - p_5) = 0 \tag{S8}$$

Note that for node 2, we would have three terms instead of two because of the three connected branches. Furthermore, for node 1, we have to account for the fluid flowing in from the pressure source $p_{\text{ext}}$, which we discuss below.

An expression for Kirchhoff's first law (i.e., the continuity equation) similar to equation (S2) can be written for every single node and a linear algebra problem can be constructed. For a network with a single pressure source as shown in Figure S1, the resulting linear algebra problem can be written in the form:

$$\begin{bmatrix} c_{12} & -c_{12} & 0 & 0 & 0 & -1 \\ -c_{12} & c_{12}+c_{23}+c_{24} & -c_{23} & -c_{24} & 0 & 0 \\ 0 & -c_{23} & c_{23}+c_{35} & 0 & -c_{35} & 0 \\ 0 & -c_{24} & 0 & c_{24}+c_{45} & -c_{45} & 0 \\ 0 & 0 & -c_{35} & -c_{45} & c_{35}+c_{45} & 1 \\ 1 & 0 & 0 & 0 & -1 & 0 \end{bmatrix} \begin{bmatrix} p_1 \\ p_2 \\ p_3 \\ p_4 \\ p_5 \\ Q_{total} \end{bmatrix} = \begin{bmatrix} 0 \\ 0 \\ 0 \\ 0 \\ 0 \\ \Delta p_{ext} \end{bmatrix} \tag{S9}$$

which we will denote as $CP = z$, where $C$ is an $n+1 \times n+1$ matrix ($n$ is the number of nodes), $P$ is an $n+1$ length vector, and $z$ is an $n+1$ length vector. There are two additional features of system (S9) to discuss. First, notice that each of the first $n$ rows correspond to an algebraic equation of a form similar to equation (S8), but the first and $n^{\text{th}}$ rows have an additional 1 or -1 in the last column which incorporates $Q_{\text{total}}$ into the equation; this occurs for the nodes that are at the inlet/outlet and connected to the pressure source. Secondly, by looking at the bottom row of the linear algebra

system (S9), we can see that an equation is specified of the form:

$$p_1 - p_n = \Delta p_{\mathrm{ext}}. \tag{S10}$$

This is exactly the condition portrayed for the placement of the pressure source in Figure S1: the pressure at node 1 is equal to the pressure at node 5 plus that of the pressure source. It turns out that an additional row needs to be added to the linear algebra problem for every pressure source ("battery") present in the network, and the entries should have the general structure portrayed here.

One issue remains. The linear algebra system (S9) corresponds to six equations but the rank of matrix $C$ is five. By "grounding" one node, we can ensure that the rank of $C$ matches the number of unknown variables in vector $P$. As illustrated in Figure S1, we intend to ground node $p_5$ (i.e., set $p_5 = 0$). This is achieved by removing the fifth column and row from matrix $C$, removing $p_5$ from vector $P$, and removing the fifth entry (0) from vector $z$. The resulting linear algebra system is:

$$\begin{bmatrix} c_{12} & -c_{12} & 0 & 0 & -1 \\ -c_{12} & c_{12}+c_{23}+c_{24} & -c_{23} & -c_{24} & 0 \\ 0 & -c_{23} & c_{23}+c_{35} & 0 & 0 \\ 0 & -c_{24} & 0 & c_{24}+c_{45} & 0 \\ 1 & 0 & 0 & 0 & 0 \end{bmatrix} \begin{bmatrix} p_1 \\ p_2 \\ p_3 \\ p_4 \\ Q_{total} \end{bmatrix} = \begin{bmatrix} 0 \\ 0 \\ 0 \\ 0 \\ \Delta p_{ext} \end{bmatrix} \tag{S11}$$

The resulting $C$ matrix still has a rank of five, and with five unknowns to be determined in vector $P$, linear algebra system (S11) can now be solved.

## 1.1 Volume Flow Rate Boundary Conditions

The example we explained above has a pressure boundary condition ($\Delta p_{ext}$) which specifies how much higher the inlet pressure $p_1$ is relative to the (grounded) outlet pressure $p_5 = 0$. Suppose we instead wanted to impose a fixed volume flow rate through the network and compute the associated pressures necessary to drive that specified flow. In Figure S1, if a known fixed volume flow rate $Q_{\mathrm{total}}$ is maintained through the network, and an unknown external pressure source $\Delta p_{\mathrm{ext}}$ adjusts so that the governing equations are satisfied. The associated linear algebra (before any grounding is applied) is as follows:

$$\begin{bmatrix} c_{12} & -c_{12} & 0 & 0 & 0 \\ -c_{12} & c_{12}+c_{23}+c_{24} & -c_{23} & -c_{24} & 0 \\ 0 & -c_{23} & c_{23}+c_{35} & 0 & -c_{35} \\ 0 & -c_{24} & 0 & c_{24}+c_{45} & -c_{45} \\ 0 & 0 & -c_{35} & -c_{45} & c_{35}+c_{45} \end{bmatrix} \begin{bmatrix} p_1 \\ p_2 \\ p_3 \\ p_4 \\ p_5 \end{bmatrix} = \begin{bmatrix} Q_{total} \\ 0 \\ 0 \\ 0 \\ -Q_{total} \end{bmatrix} \tag{S12}$$

We then ground node 5 (i.e., set $p_5 = 0$) by removing the fifth row and column of matrix $C$, removing $p_5$ from vector $P$, and removing the fifth entry of vector $z$, leaving:

$$\begin{bmatrix} c_{12} & -c_{12} & 0 & 0 \\ -c_{12} & c_{12}+c_{23}+c_{24} & -c_{23} & -c_{24} \\ 0 & -c_{23} & c_{23}+c_{35} & 0 \\ 0 & -c_{24} & 0 & c_{24}+c_{45} \end{bmatrix} \begin{bmatrix} p_1 \\ p_2 \\ p_3 \\ p_4 \end{bmatrix} = \begin{bmatrix} Q_{total} \\ 0 \\ 0 \\ 0 \end{bmatrix} \quad \text{(S13)}$$

Thus, after solving this linear algebra system, the pressure difference (relative to $p_5 = 0$) required to drive a volume flow rate specified by $Q_{total}$ is given by the obtained value of $p_1$.

## 1.2 Mixed Boundary Conditions

The liver is anatomically unique in that it has two inlet vessels: the hepatic artery (at a fixed pressure) and the portal vein (at a fixed volume flow rate). An idealized network with these two different inlet boundary conditions is shown in Figure S2.

The linear algebra that specifies the network shown in Figure S2 can be written as:

$$\begin{bmatrix} c_{02} & 0 & -c_{02} & 0 & 0 & 0 & 0 \\ 0 & c_{12} & -c_{12} & 0 & 0 & 0 & -1 \\ -c_{02} & -c_{12} & c_{02}+c_{12}+c_{23}+c_{24} & -c_{23} & -c_{24} & 0 & 0 \\ 0 & 0 & -c_{23} & c_{23}+c_{35} & 0 & -c_{35} & 0 \\ 0 & 0 & -c_{24} & 0 & c_{24}+c_{45} & -c_{45} & 0 \\ 0 & 0 & 0 & -c_{35} & -c_{45} & c_{35}+c_{45} & 1 \\ 0 & 1 & 0 & 0 & 0 & -1 & 0 \end{bmatrix} \begin{bmatrix} p_0 \\ p_1 \\ p_2 \\ p_3 \\ p_4 \\ p_5 \\ Q_{total} \end{bmatrix} = \begin{bmatrix} Q_2 \\ 0 \\ 0 \\ 0 \\ 0 \\ -Q_2 \\ \Delta p_{ext} \end{bmatrix} \quad \text{(S14)}$$

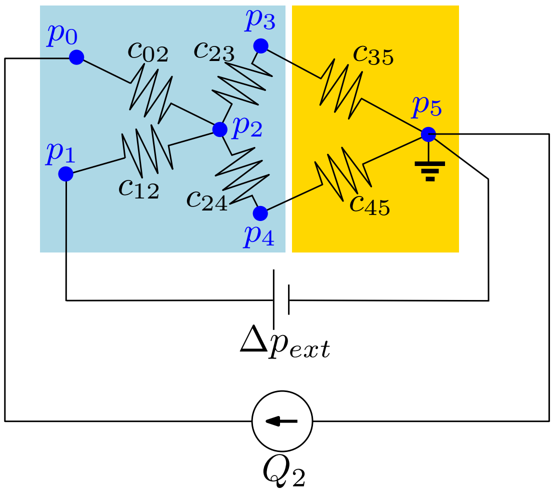


**Fig. S2** A two-inlet hydraulic network model with different boundary conditions (one pressure, one volume flow rate). The pressure boundary condition ($\Delta p_{ext}$) is analogous to the hepatic artery and the volume flow rate boundary condition ($Q_2$) is analogous to the portal vein.

Again, grounding node 5, this linear algebra system can be re-written as:

$$\begin{bmatrix} c_{02} & 0 & -c_{02} & 0 & 0 & 0 \\ 0 & c_{12} & -c_{12} & 0 & 0 & -1 \\ -c_{02} & -c_{12} & c_{02}+c_{12}+c_{23}+c_{24} & -c_{23} & -c_{24} & 0 \\ 0 & 0 & -c_{23} & c_{23}+c_{35} & 0 & 0 \\ 0 & 0 & -c_{24} & 0 & c_{24}+c_{45} & 0 \\ 0 & 1 & 0 & 0 & 0 & 0 \end{bmatrix} \begin{bmatrix} p_0 \\ p_1 \\ p_2 \\ p_3 \\ p_4 \\ Q_{total} \end{bmatrix} = \begin{bmatrix} Q_2 \\ 0 \\ 0 \\ 0 \\ 0 \\ \Delta p_{ext} \end{bmatrix} \quad \text{(S15)}$$

Here, the total volume flow rate out through node 5 is $Q_{\text{total}} = Q_1 + Q_2$ where $Q_1$ is the volume flow rate through the "battery".

### 1.3 Collateral Pathways

Since the volume flow rate through the portal vein which we specified in the main text (based on clinical measurements) *excludes* collateral flow, we solve a linear algebra system of a form analogous to system (S15). We then compute the collateral volume flow rate as $Q_{col} = c_{col}\Delta p_{PV}$, where $c_{col}$ is the collateral conductance and $\Delta p_{PV}$ is the portal vein inlet pressure relative to the vena cava, analogous to $p_0$ in system (S15).

### 1.4 Sparse matrix representation for computational tractability

It is important to note that for large networks, the matrix $C$ is *sparse*, meaning it contains mostly zeros. It is not computationally efficient to store all these zeros, and when very large networks are modeled, it can become prohibitively expensive to do so. In such cases, it is instead necessary to create a sparse 2D array for C. In MATLAB, this can be achieved by using the function "sparse."

## 2 Results based on lumped sinusoid channels with a Gaussian distribution

In Section 3, we discuss the effects of change in lumped sinusoid channel conductance with diameter at a given sinusoid conductance $c_{sin}$ and present results with varying levels of sinusoid heterogeneity $\sigma$ in each Couinaud segment for models of a healthy liver, early cirrhosis, and advanced cirrhosis. The distribution of conductance of the lumped sinusoid channels in each segment were generated using a log-normal distribution with a fixed mean and standard deviation. We also explored an alternative distribution in which the lumped sinusoid diameter $d$ was modeled using a Gaussian distribution. Since exponentiation and multiplicative scaling of a log-normal distribution is still log-normal, one may interpret the results in the main text as based on a log-normal distribution of lumped sinusoid diameter or conductance. However, exponentiation of a Gaussian distribution results in a distribution that is *not* Gaussian. Thus, the results presented here correspond to a Gaussian distribution in lumped sinusoid diameter $d$, not conductance.

In the following section, we characterize the distribution of lumped sinusoid conductance values and generate plots of volume flow rate through the liver, as well as the inlet HA and PV pressure differences for different cirrhosis stages. We also present segmental volume flow rate, perfusion and fold change in WSS based on analogous

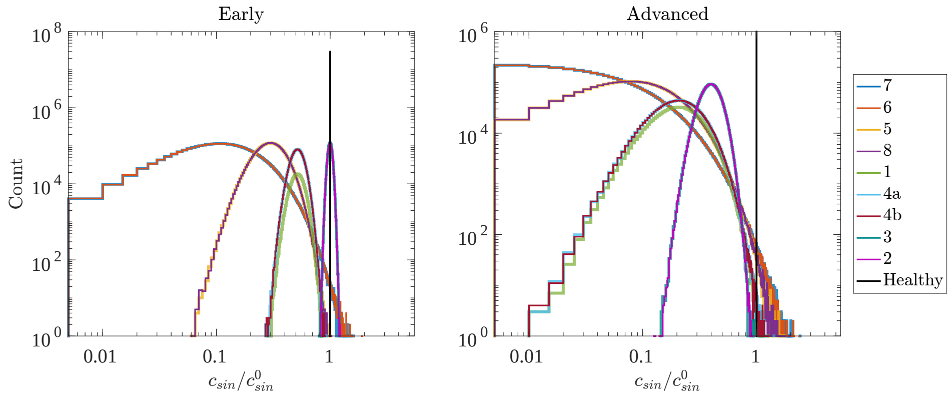


**Fig. S3** Distribution of lumped sinusoid channel conductance based on Gaussian-sampled lumped sinusoid diameter $d$ for livers with early cirrhosis (left) and advanced cirrhosis (right) for all nine Couinaud segments.

segment-wise parameters with a Gaussian distribution. These simulations were generated using the same parameters listed in Table 3 (with lumped sinusoid diameter related to conductance by equation (1) in the main text). The results indicate that both distributions produce results that are qualitatively similar and in good agreement with clinical observations. Hence, further experiments are required to determine which distribution most accurately captures sinusoid anatomy.

Figure S3 shows distributions of lumped sinusoid conductance generated based on Gaussian distributions of lumped sinusoid diameter $d$ for a healthy liver, early cirrhosis, and advanced cirrhosis. Negative values generated by the Gaussian distribution were assigned a very small conductance value, corresponding to an essentially closed channel. The plots show a broadening distribution and a leftward shift in peak across all segments, as heterogeneity increases, which is consistent with the trends observed in the log-normal distribution, as seen in Figure 5 in the main text.

Figure S4 plots the volume flow rate and inlet HA and PV pressure differences for different stages of cirrhosis, when the conductance of the lumped sinusoid channels are generated based on a Gaussian distribution of $d$. Similar to Figure 7 in the main text, the PV volume flow rate decreases for a cirrhotic liver (compared to the healthy liver), but remains constant between early and advanced cirrhosis stages. HA flow declines slightly with cirrhosis progression, RHV flow decreases rapidly, MHV flow decreases slightly, and LHV flow increases, indicating right lobe atrophy and left lobe hypertrophy, consistent with trends seen in the liver model generated with a log-normal distribution. The non-uniform redistribution across hepatic veins, indicating right lobe atrophy and left lobe hypertrophy are also seen in Figure S5, which plots the fraction of total volume flow rate through various inlet and outlet vessels in the healthy liver, early cirrhosis, and advanced cirrhosis.

Plots of segmental volume flow rate and perfusion for a healthy, early cirrhotic, and advanced cirrhotic liver are shown in Figure S6, and plots of fold change in WSS are presented in Figure S7. These plots show preferential routing of blood and increased left and right lobe polarity as cirrhosis progresses. The plots show minor differences

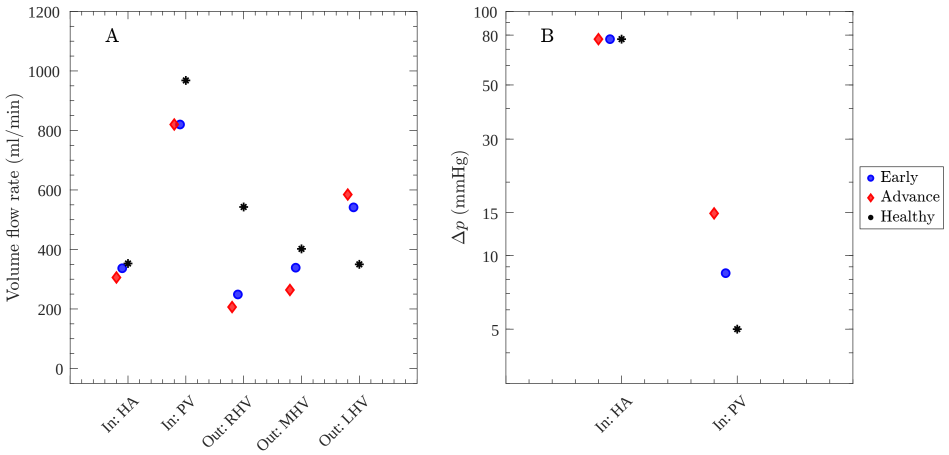


**Fig. S4** Plots of volume flow rate and inlet pressure differences (relative to the vena cava) when lumped sinusoid channel diameters are generated using a Gaussian distribution. (A) Volume flow rate through HA, PV, RHV, MHV, and LHV, and (B) pressure differences in HA and PV for healthy, early cirrhotic, and advanced cirrhotic livers. In all cases, $c_{col}/c_{liver} = 0.01$.

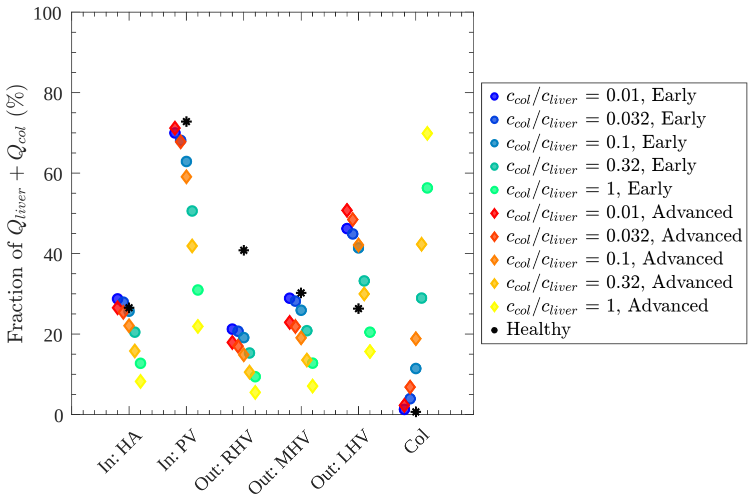


**Fig. S5** Fraction of total volume flow rate through liver and collateral ($Q_{liver}+Q_{col}$) passing through HA, PV, RHV, MHV, LHV, and collateral when lumped sinusoid channel diameters are generated using a Gaussian distribution. Note that for a given marker, HA, PV, and Col sum to 100%, and RHV, MHV, LHV, and Col sum to 100%.

in flow rates between results based on lumped sinusoid channels with a Gaussian distribution compared to a log-normal distribution, but are consistent in the overall trends they exhibit.

In summary, lumped sinusoid channels generated with both Gaussian and log-normal distributions of $d$ show right lobe atrophy and left lobe hypertrophy, realistic portal pressures, and volume flow rates that are in line with clinical observations.

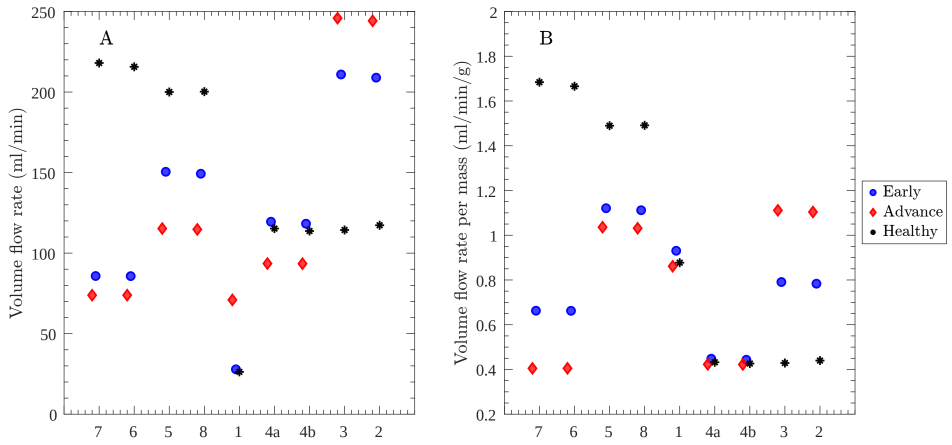


**Fig. S6** Plots of (A) volume flow rate and (B) perfusion in individual Couinaud segments for a healthy, early cirrhotic, and advanced cirrhotic liver when lumped sinusoid channel diameters are generated using a Gaussian distribution. In all cases, $c_{col}/c_{liver} = 0.01$.

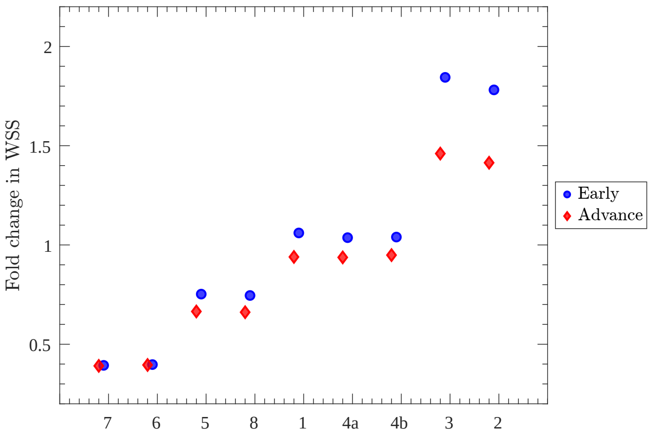


**Fig. S7** Plots of mean fold change in WSS for a healthy, early cirrhotic, and advanced cirrhotic liver when lumped sinusoid channel diameters are generated using a Gaussian distribution. In all cases, $c_{col}/c_{liver} = 0.01$.

Therefore, further experiments are required to conclusively determine which distribution (and parameters) most accurately captures true liver anatomy and the alterations that occur as cirrhosis progresses.
